# Dating the megalithic culture of laos: Radiocarbon, optically stimulated luminescence and U/Pb zircon results

**DOI:** 10.1371/journal.pone.0247167

**Published:** 2021-03-10

**Authors:** Louise Shewan, Dougald O’Reilly, Richard Armstrong, Phillip Toms, John Webb, Nancy Beavan, Thonglith Luangkhoth, Jamie Wood, Siân Halcrow, Kate Domett, Julie Van Den Bergh, Nigel Chang

**Affiliations:** 1 School of Earth Sciences, University of Melbourne, Victoria, Australia; 2 School of Historical and Philosophical Studies, University of Melbourne, Victoria, Australia; 3 School of Archaeology and Anthropology, Australian National University, Canberra, Australia; 4 Research School of Earth Sciences, Australian National University, Canberra, Australia; 5 Luminescence Dating Laboratory, University of Gloucestershire, Cheltenham, United Kingdom; 6 Department of Ecology, Environment and Evolution, La Trobe University, Melbourne, Australia; 7 Institute of Environmental Science and Research Ltd, Porirua, New Zealand; 8 Department of Heritage, Ministry of Information Culture and Tourism, Vientiane, Lao PDR; 9 Luminescence Dating Laboratory, University of Gloucestershire, Cheltenham, United Kingdom; 10 Department of Anatomy, University of Otago, Dunedin, New Zealand; 11 College of Medicine and Dentistry, James Cook University, Townsville, Australia; 12 Lamma Island, Hong Kong, China; 13 College of Arts, Society and Education, James Cook University, Townsville, Australia; Universita degli Studi di Milano, ITALY

## Abstract

The megalithic jar sites of Laos (often referred to as the Plain of Jars) remain one of Southeast Asia’s most mysterious and least understood archaeological cultures. The sites, recently inscribed as UNESCO World Heritage, host hollowed stone jars, up to three metres in height, which appear scattered across the landscape, alone or clustered in groups of up to more than 400. Until now, it has not been possible to estimate when the jars were first placed on the landscape or from where the stone was sourced. Geochronological analysis using the age of detrital zircons demonstrates a likely quarry source for one of the largest megalithic jar sites. Optically Stimulated Luminescence (OSL) dating suggests the jars were positioned at the sites potentially as early as the late second millennium BC. Radiocarbon dating of skeletal remains and charcoal samples places mortuary activity around the jars from the 9-13th century AD, suggesting the sites have maintained ritual significance from the period of their initial placement until historic times.

## Introduction

Northern Laos is home to one of Southeast Asia’s most enigmatic archaeological cultures. The megalithic jar sites of Laos comprise one to three-metre-tall carved stone jars dotted across the landscape, appearing alone or in groups of up to several hundred. The majority of these sites are found in Xieng Khouang Province, and while collectively termed the ‘Plain of Jars’, the sites are mostly located on mountain ridges, saddles or hill slopes surrounding the central plain and upland valleys. While brought to the attention of Western scholars in the late 1800s [1], it was not until the pioneering expeditions by Madeleine Colani (1866–1943), of the École française d’Extrême-Orient (EFEO), that significant research on the megalithic sites commenced [2,3]. Since that time, limited research has been conducted on these megalithic sites owing mostly, to the presence of unexploded ordnance (UXO). Of the more than 100 identified jar sites, less than 10% have been cleared and are accessible for ‘traditional’ archaeological investigation.

In recent years archaeological research conducted by the authors has resulted in the excavation of selected areas at three jar sites and broader surveys of the region, increasing our knowledge of the sites and their enduring ritual significance [4–8]. The three excavated sites (1, 2, and 52) form part of a group of 11 sites (Sites 3, 8, 12, 21, 23, 25, 28 and 42) inscribed in 2019 on the UNESCO World Heritage list (https://whc.unesco.org/en/list/1587/). This recent research identified varied mortuary practices at Site 1 (primary interment, secondary interment of bundled bone, and bone in ceramic jars) and similar evidence for burial markers at the sites 2 and 52 although human bone was not recovered at these latter two sites, likely due to taphonomic processes. How the burials uncovered at Site 1 relate to the megaliths is unclear as the timing of the placement of the jars has, until now, proven difficult to estimate.

In this paper we start by briefly outlining what is known about the sites based on limited previous research and more recent excavation and research conducted by the authors. Many questions remain however, regarding the megaliths of Laos such as, the date when the megaliths were placed in their current location (and the chronological relationship between mortuary activity and the jars) and the likely provenance of the stone used to create the jars at Site 1.

To establish a chronological framework for the sites and mortuary activity we present radiocarbon dates from Site 2 (none were viable for Site 52), and compare these with those previously obtained from Site 1 [6]. In an effort ascertain when the megaliths were placed in the landscape, sediment samples beneath the megaliths at Site 2 and Site 52 were subjected to Optically Stimulated Luminescence (OSL) dating. To determine the likely provenance of the stone used to create the jars at Site 1, we used U-Pb zircon dating. While geologists have used this method for several decades, this approach has become increasingly common in archaeological provenance studies [9–12]. In this study we compared the ages of zircons present in sandstone from a jar at Site 1 with two samples of sandstone from the likely quarry source (Site 21, Phoukeng).

### Sites investigated and previous research

This paper focuses on research conducted at four megalithic jar sites; 1, 2, 52 and one quarry site (Site 21). The sites are located on and around the Plain of Jars, which is an ~80 km2 alluvial plain at an elevation of ~1100 m asl, surrounded by forested mountains of Palaeozoic sediments that rise up to ~2000 m asl to the south. This plain is a natural grassland as the soils have low fertility (acidic and low in N and P) and cannot support a forest cover; the vegetation is dominated by a single grass species (Themeda *triandra)* [13].

#### Site 1

Site 1 (N 19°25ʼ48′′ E 103°9ʼ18′′), located 4 km southwest of the capital of Xieng Khouang Province, Phonsavan, on the eastern edge of the plain comprises over 300 jars, several discs and unmodified boulders, distributed in five groups. The site is dominated by a limestone outcrop rising to a crest at 1125 m asl. Further east, hills of Palaeozoic sediments rise to over 1200 m asl. In parts, Site 1 is underlain by the white—pale cream fine-grained alluvial sediments while in other areas the soil is reddish-brown beneath a brown-pale brown layer.

**Fig 1:**
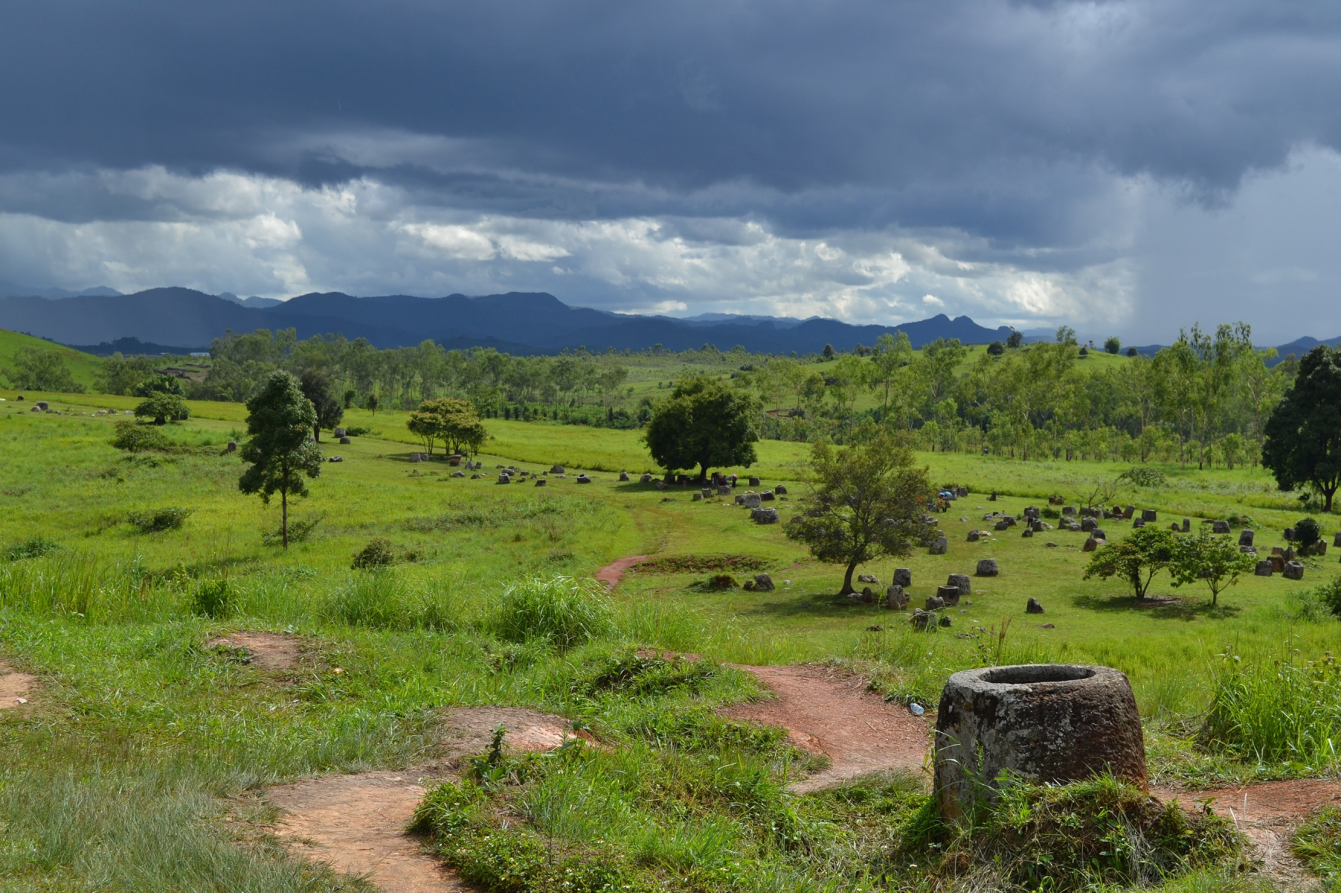
View to the southwest at megalithic jar Site 1.

Colani excavated at Site 1 (Fig 1), and believed the sites dated to the Southeast Asian Iron Age (c. 500 BC to 500 AD) based on associated material culture [2,3]. Colani recovered fragmented skeletal remains and material culture including glass and carnelian beads, ceramic vessels (including burial jars), ear discs, spindle whorls, iron and bronze tools and jewelery and ground stone artifacts. Subsequent excavations by Nitta [14], Sayavongkhamdy [15] and later rescue excavations revealed several burial contexts and material culture similar to that found by Colani. The two latter investigations also produced a series of radiocarbon dates for activity around the stone jars ranging from 7577–7079 calBC and 1027–1220 calAD (S1 Table) [15].

**Fig 2:**
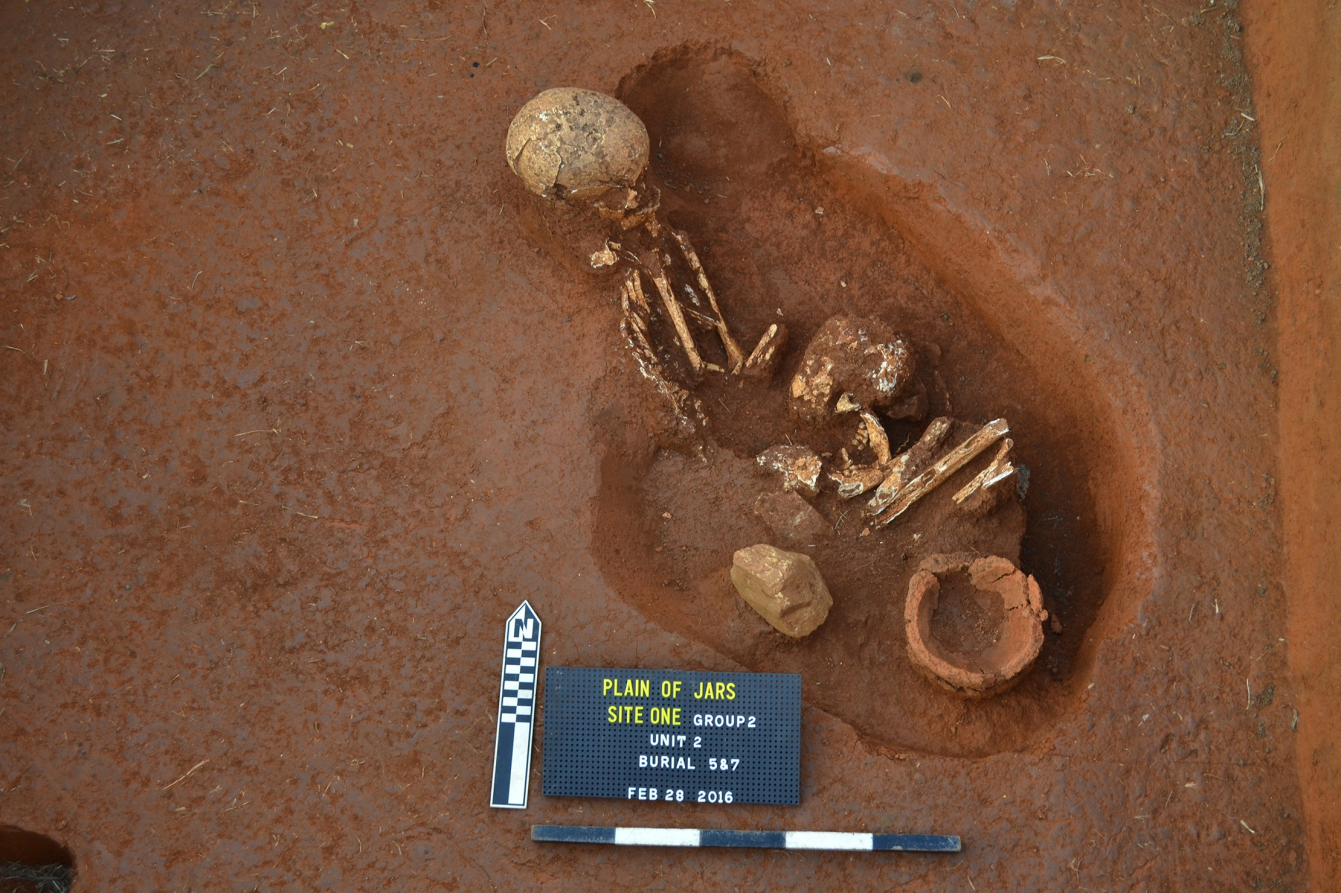
Primary interment, Burial 5 & 7 at Site 1.

**Fig 3:**
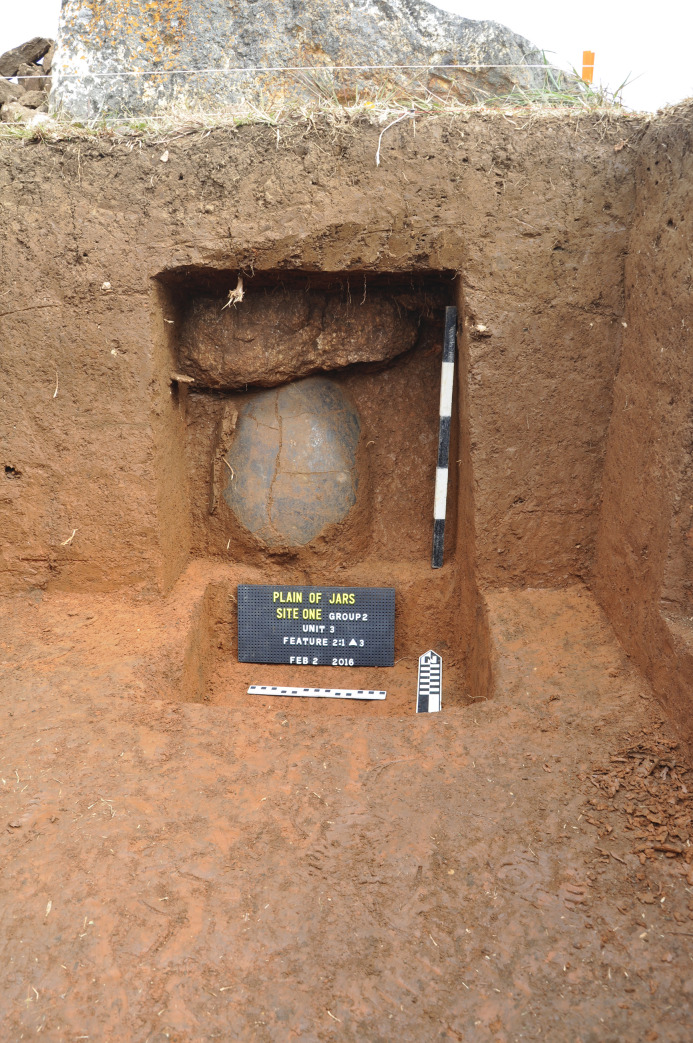
Ceramic burial jar beneath quartz rich sandstone boulder at Site 1.

**Fig 4:**
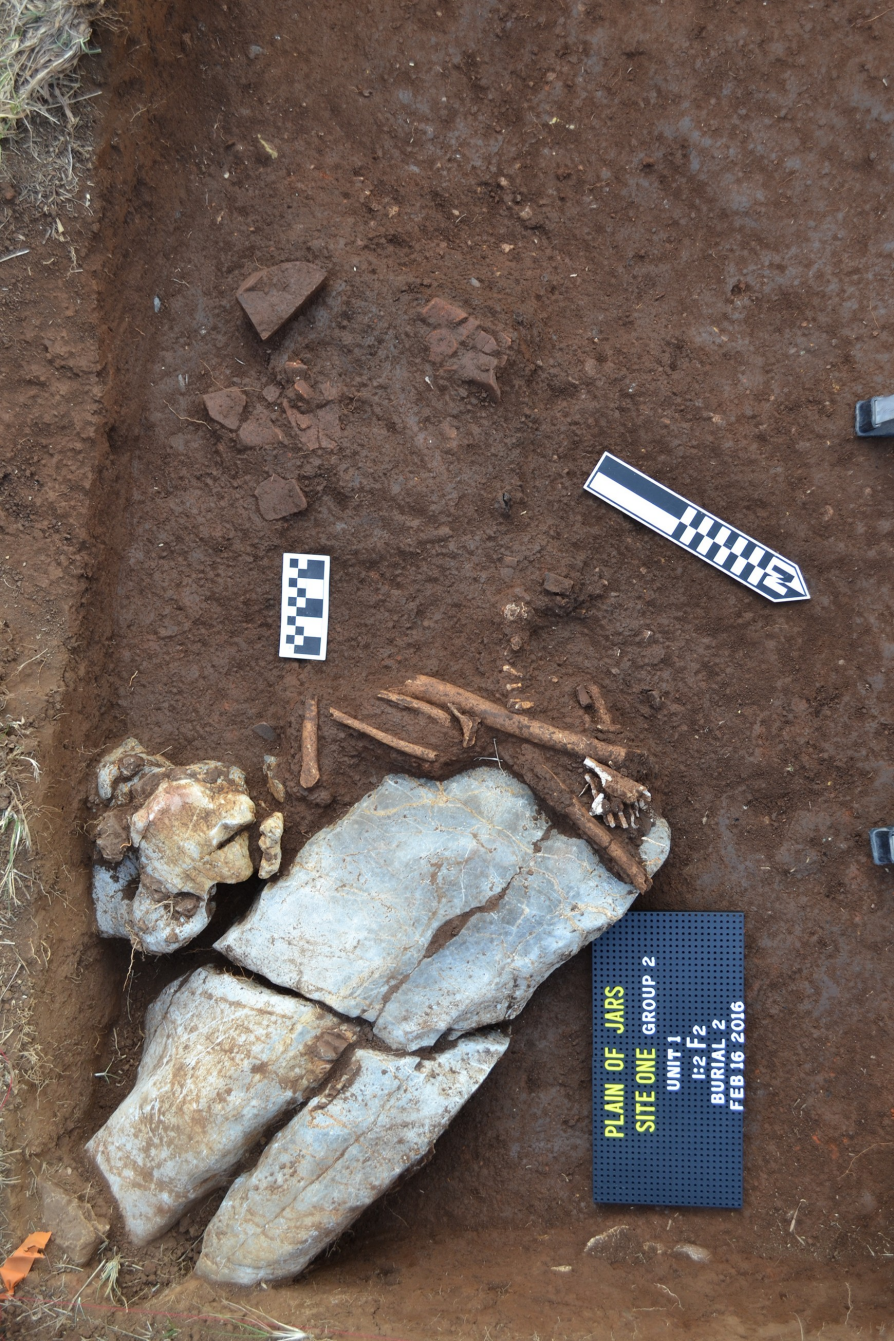
Burial 2, Site 1. A secondary burial with bones associated with limestone blocks and beneath a sandstone disc (disc removed in photo).

Three units were excavated in 2016 at Site 1, all located amongst the megalithic jars [4–6]. The most notable aspect of the excavations was the discovery of divergent mortuary practice including primary interment and two forms of secondary burials comprising the placement of human remains in ceramic vessels and bundles of interred skeletal material (Figs 2–4). The three excavation units produced a minimum number of 18 individuals including a large proportion (>60%) of subadults (younger than 15 years of age). Other remarkable features included rough pavements of sandstone chips, buried limestone boulders and slabs (one of which was perforated and placed over the skull and torso of an interred individual), siliceous quartz breccia boulders which are associated with the mortuary ritual, and a range of cultural artefacts. The archaeological deposits were shallow, terminating at between c. 50–60 cm below surface (b.s.) across all excavated units, with no evidence for residential activity. Thirty-two charcoal samples (from both mortuary and level contexts) and two bone samples were subjected to radiocarbon dating analysis (S2 Table).

The 316 megaliths at Site 1 are fashioned from sandstone and conglomerate (84% are sandstone) and range up to c. 2.5 m in height. The closest quarry locale to Site 1 and the presumed source of the jars, based on the presence of incomplete jars and similarity in stone type and treatment, is known as Site 21 (Phoukeng), located c. 8km to the northwest. To substantiate this supposition, the authors compared fragments from a broken jar at Site 1 with samples of stone and incomplete jars acquired from Site 21 and subjected them to U-Pb dating.

#### Site 2

**Fig 5:**
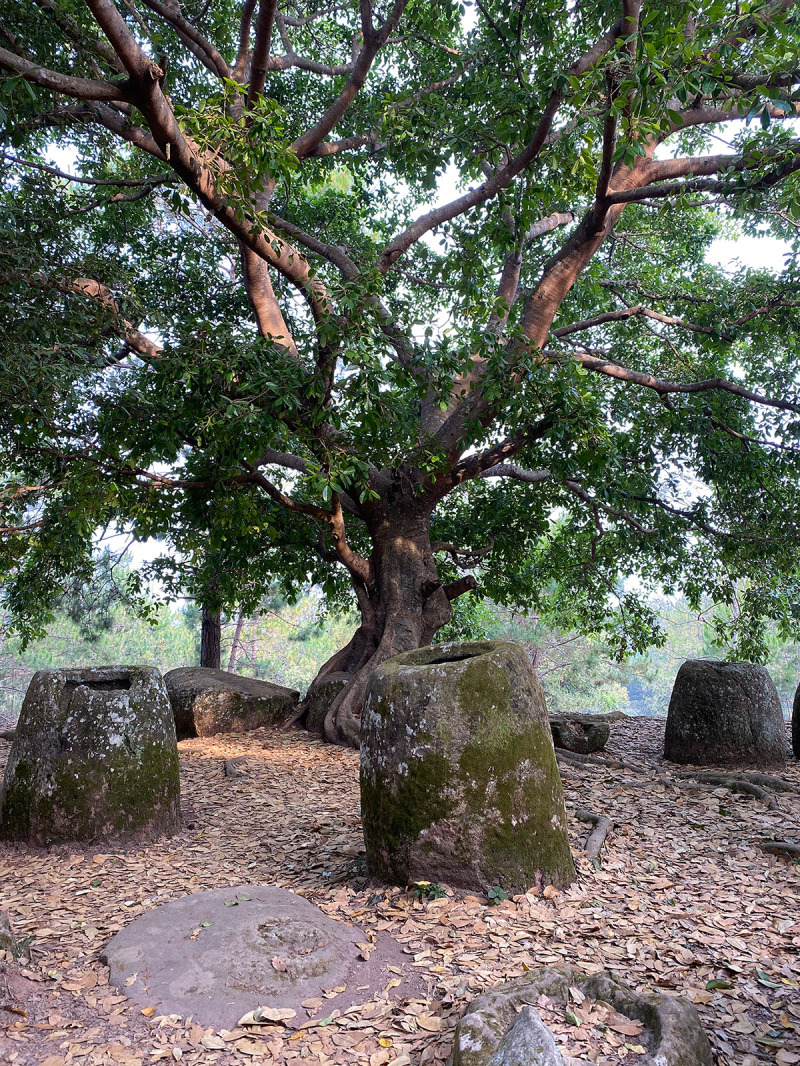
View of megalithic jars at Site 2.

Site 2 (N 19°19ʼ12′′ E 103°9ʼ15′′) is located approximately 15km south of Phonsavan on the edge of the central plain and contains 86 sandstone jars and 15 discs distributed over two knolls (Fig 5). Site 2 is located on the crest of a ridge at an elevation of ~1150 m asl; this ridge forms part of the southern catchment boundary of the plain, with uninterrupted views across the plain to the north. The site is underlain by fine-grained Palaeozoic redbed sediments, which give the soil a distinctive pale reddish colour. These sediments are identified as the Permian Khang Khai formation on the Khangkhai 1:200,000 geological map. Examination of the soil shows that it is composed of fine quartz silt (5–15 μm) with a component of clay and iron oxide.

Colani [2,3] undertook excavation around the megalithic jars at Site 2 finding ceramic vessels, spindle whorls, earrings, ceramic weights, bronze jewellery and bells, glass and carnelian beads, stone pendants, stone adzes, grinding stones and iron knives. No human bone was recorded [3]. In 1996, test-pit excavations at Site 2 were conducted by Sayavongkhamdy [15] which uncovered similar artefacts to those documented by Colani and one radiocarbon date (AA81046 see S1 Table) was recovered during the period of UXO clearance at the site in 2008.

Three excavation units, located among the megaliths on the western knoll, were excavated by the authors in 2019. Excavations revealed similar material culture and features to that found at Site 1 [6]. As at Site 1, purposefully placed limestone slabs were found at Site 2, however, unlike Site 1 there was no associated human bone. This might signify different taphonomic processes at Sites 1 and 2. Sixteen charcoal samples were subjected to radiocarbon dating analysis and sediment samples from beneath two stone jars were taken for OSL analysis.

#### Site 52

**Fig 6:**
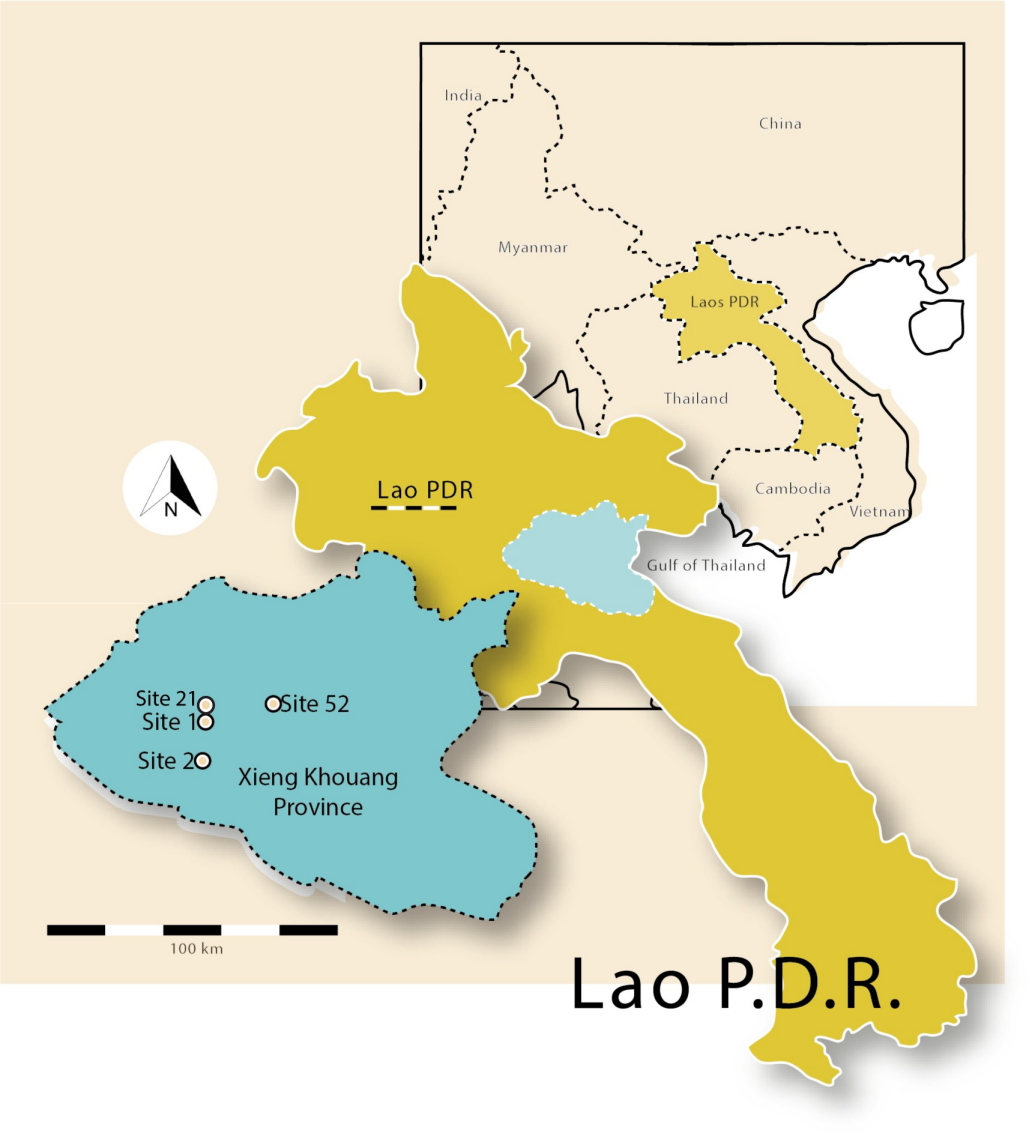
Map of Laos displaying Xieng Khouang Province, sites excavated and quarry, Site 21.

At Site 52 (N 19°29ʼ42′′ E 103°25ʼ56′′), located in mountainous terrain c. 25 km northeast of Phonsavan, 415 megalithic stone jars and a collection of 219 discs, lids and burial-marker boulders are scattered across a saddle on a ridge crest at approximately 1200 m asl (Fig 6). The area contains fine-grained red Palaeozoic sandstone outcrops with variable degrees of weathering and natural variations in the depositional environments. Limestone forms the erosion-resistant cappings on ridges in the region and appears as boulder float near the site. The soil at site 52 is red in colour, similar to that at Site 2.

**Fig 7:**
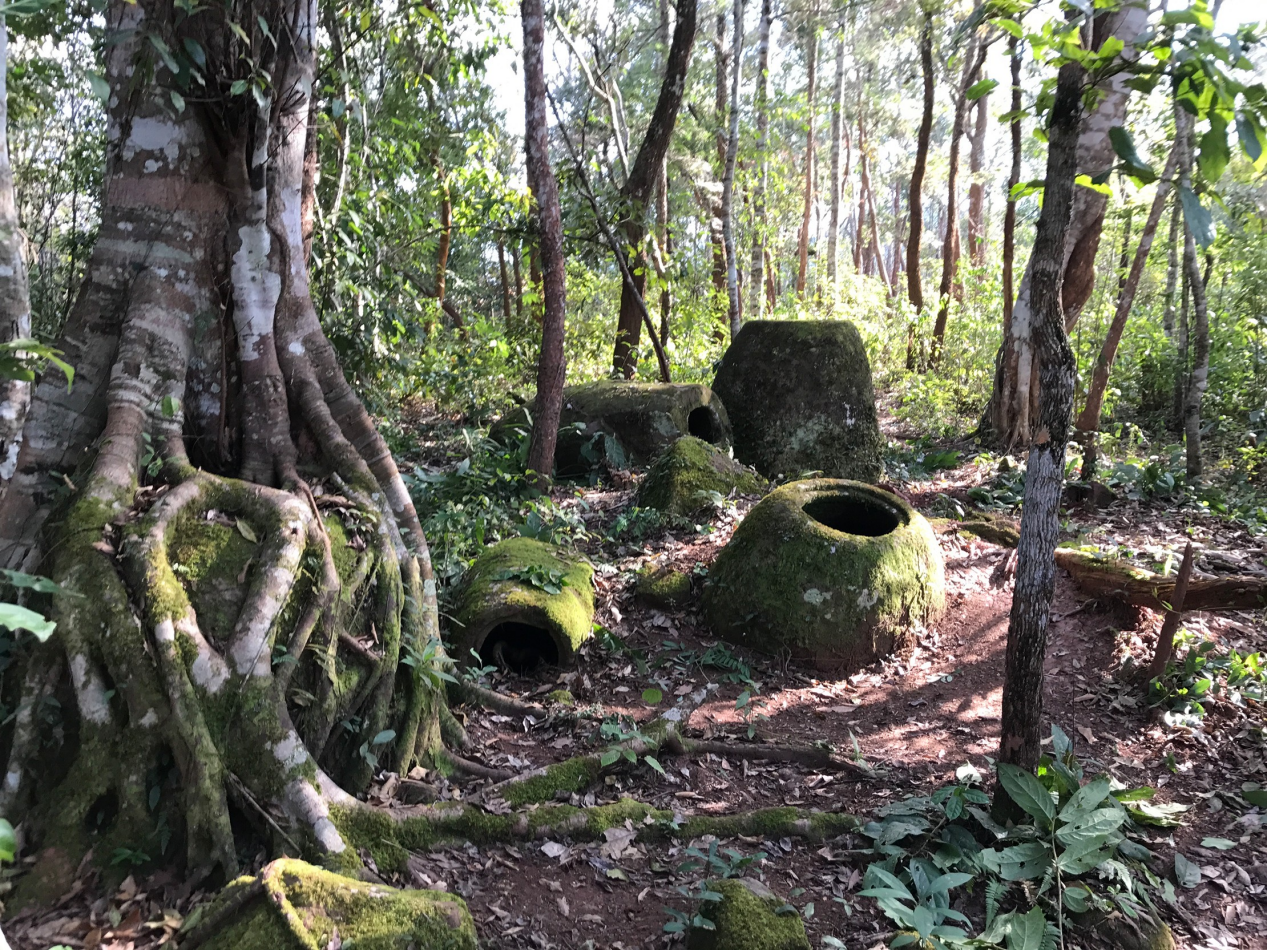
View of megalithic jars at Site 52.

Site 52 was discovered in 2005, with survey and inventory conducted in 2008. In 2017, the authors excavated eight units of varied dimensions amongst the jars (Fig 7) [7]. The site had a paucity of artefacts but features similar to Sites 1 and 2, such as the presence of limestone slabs and sandstone chip pavements, were found. Other than a single dental specimen, no skeletal material was identified and no suitable samples were obtained for radiocarbon dating. Sediment samples from beneath two stone jars were taken for OSL dating.

#### Site 21

**Fig 8:**
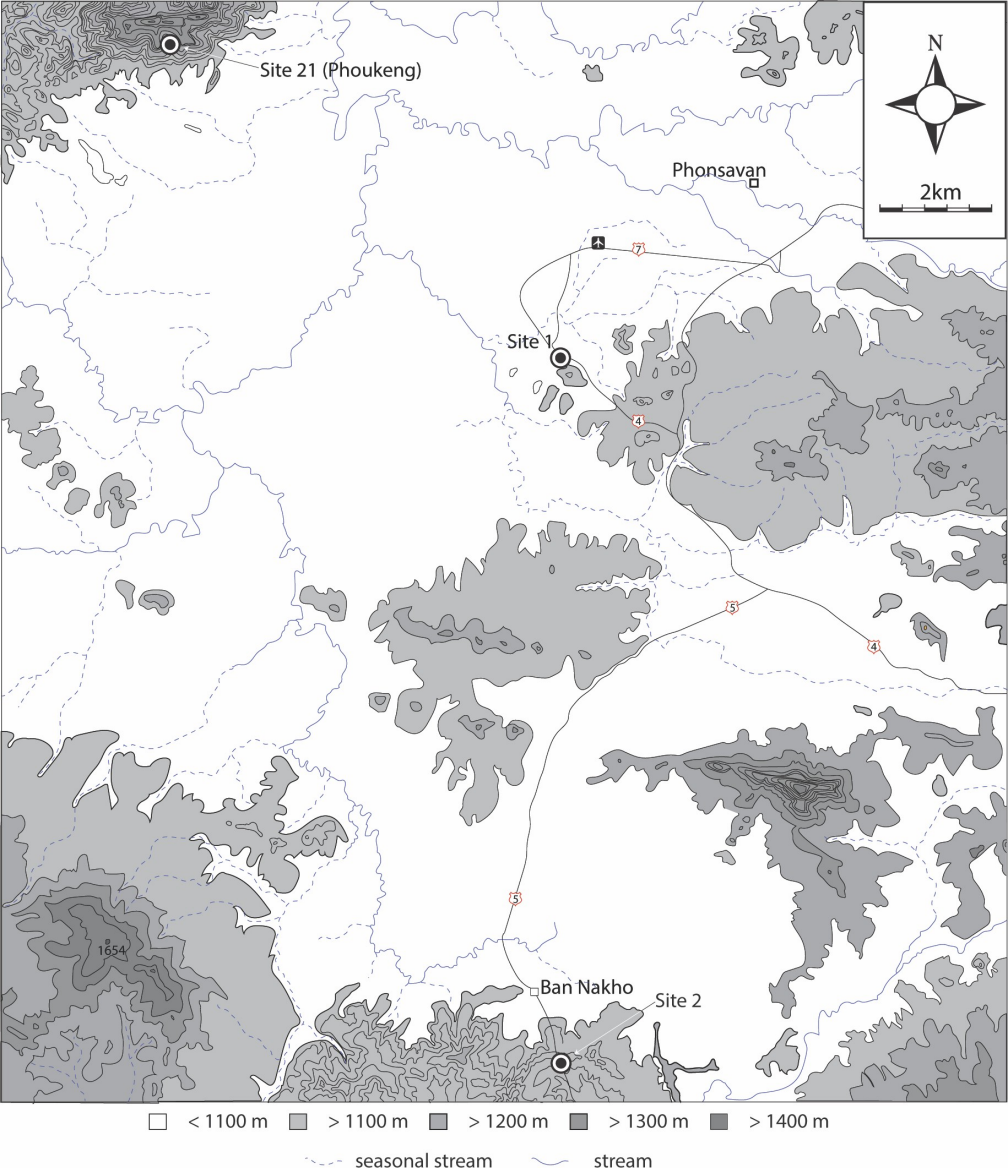
Map showing locations of Sites 21 (Phoukeng Quarry), 1 and 2, Xieng Khouang Province.

Site 21 (N 19°28’50” E 103°05’15”), c. 8 km from Site 1 has been identified as a quarry site and may have been the source of stone for the jars at Site 1 (Fig 8). The quarry, located on the slopes of a mountain that rises to over 1400 m asl, is expansive (approximately 20 ha) with the remains of stone jars in various stages of production, from complete to basic rough out. The site is heavily contaminated with UXO and not accessible for excavation, however, rock samples for U-Pb dating were retrieved.

## Materials and methods

Through the combination of radiocarbon dating of charcoal and bone, OSL dating of sediments and U-Pb dating of zircons within the stone from which the jars are made, we have sought to expand our knowledge regarding the provenance of the stone from which the megaliths are fashioned, the timing of their placement and the relationship between the megaliths and the surrounding mortuary activity. Permits to work at the megalithic sites were obtained from the Lao Department of Heritage, Ministry of Information Culture and Tourism, Lao PDR.

### Archaeological excavation

At Site 1, excavations in 2016 were conducted over three units [4,6]. In 2017, eight units were dug at Site 52 [7] and in 2019 three units were opened at Site 2. Excavation was undertaken in arbitrary 10 cm spits by layer. Layer numbers were changed when significant variation in soil colour was noted and material was screened using a 5 mm sieve.

### Radiocarbon dates

Charcoal samples from Sites 1 and 2 were submitted to the Australian National University (ANU), Canberra. After physically cleaning individual fragments with a scalpel, charcoal was pretreated using an acid-base-acid (ABA) protocol. For bone samples, collagen was extracted and purified according to an ultrafiltration protocol. An HCl solution was used to remove the bone mineral and exogenous carbonates, and alkali was used to remove humic substances. The sample was then gelatinized by heating in a weak HCl solution. The resulting soluble gelatin allows for larger insoluble particles to be removed with a pre-cleaned filter, followed by a second filtering with a pre-cleaned >30K Dalton molecular weight cutoff size ultrafilter to remove smaller-sized exogenous material [16]. Both bone and charcoal samples were prepared for graphitisation by combustion in a sealed quartz tube, and graphitised over an iron catalyst with hydrogen gas before measurement in a Single Stage AMS at the ANU [17]. Dates have been calculated according to Stuiver and Polach using δ^13^C measured by AMS [18]. Calibrated radiocarbon ages obtained by previous researchers at Site 1 are presented in S1 Table and dates for samples collected by the authors at Site 1 in 2016 are presented in S2 Table. The dates are presented at 95.4% confidence using OxCal v.4.4 and the IntCal 20 calibration curve [16,19,20].

### OSL

In an effort to establish a *terminus post quem* for jar emplacement, OSL samples were taken from sediments underneath two jars at Site 52 in 2017 (052030064 located at N19.49553698 E103.4319365 and 052020052 located at N19.49593697 E103.4328666) and two jars at Site 2 in 2019 (W0013 located at N19.31881937 E103.15284144 and W0021 located at N19.31880992 E103.15278109). Such dating provides an estimate of when sediment was last exposed to light, which, in near surface sediments, will reflect the age of primary sediment deposition or cessation of reworking [21,22] or a blend of the two. The OSL samples collected at Site 2 were obtained by horizontally hammering 20 cm lengths of 35 mm (diameter) opaque plastic tubing directly into the sediment. Samples were obtained immediately beneath each of the jars and at ~10 cm intervals vertically in order to assess the consistency of age with relative stratigraphic position. At Site 52 samples were removed in total darkness from beneath the megaliths using a trowel and sealed in light-proof boxes.

Owing to the clayey-silt texture of the deposits, quartz in the fine silt (5–15 μm) fraction was isolated; this involved acid and alkaline digestion (10% HCl, 15% H_2_O_2_), followed by sedimentation in acetone, a further acid digestion (35% H_2_SiF_6_ for 2 weeks; [23,24] and then an acid wash (10% HCl). Twelve multi-grain aliquots (~1.5 mg) were then mounted on aluminium discs for Equivalent Dose (D_e_) evaluation using the Single-Aliquot, Regenerative-Dose (SAR) protocol [25,26] and a Risø TL-DA-15 irradiation-stimulation-detection system [27,28]. Dose Recovery tests [26] were used to establish preheat treatments and, in part, to qualify the reliability of D_e_ values. Repeat regenerative-doses [25] were used to quantify repeat ratios and thus success in correction of signal sensitisation induced by the measurement sequence. Post-IR OSL ratios [29] were used to confirm the absence of significant feldspar contamination. Geometric mean D_e_ values for each sample were calculated using the Central Age Model [30]; the application of Minimum Age or Finite Mixture Models [31,32] is not appropriate here given the size and number of grains per aliquot when OSL dating fine silt. Lithogenic concentrations of U, Th, K and confirmation of negligible U-disequilibrium were established using laboratory-based γ spectrometry: an Ortec GEM-S high purity Ge coaxial detector system. Radionuclide concentrations were converted into dose rate (D_r_) values [33], accounting for D_r_ modulation forced by grain size [34], present moisture content [35] and reduced signal sensitivity to α radiation (a-value 0.050 ± 0.002).

To assess the influence of jar material on the gamma dose rate within 15cm of the sediment-artefact interface, a basal fragment of jar 052020052 at Site 52 (above sediment sample GL17084) was collected. The geometry of the jar’s base (c. 0.6 m diameter, c. 0.2 m thick) suggests c. 50% of the gamma radiation field interacting with the OSL sample immediately beneath would be located within the jar. The fractional gamma dose rate function in the R Luminescence package [36,37] was used to calculate the gamma dose rate for sample GL17084. Cosmogenic D_r_ values were calculated on the basis of sample depth, geomagnetic latitude and matrix density [38]. Age estimates are based on the quotient of D_e_ and D_r_ and expressed relative to the year of sampling. Since the number of grains in the fine silt aliquots is many magnitudes greater than a fine sand aliquot, there is almost no overdispersion/difference in age between aliquots, and overdispersion in the data is not a problem.

### Zircon geochronology

U-Pb dating of zircon has long been a preferred and reliable method for establishing igneous and metamorphic ages of crustal (felsic) and—in the rare instances where zircons exist–in mantle (mafic) rocks. Zircon is the ideal mineral for U-Pb dating as it includes significant amounts of uranium but, crucially, excludes initial Pb in the original structure. The decay of the U to radiogenic Pb through two separate decay schemes (^238^U to ^206^Pb, half-life 4460 Ma; ^235^U to ^207^Pb, half-life 700 Ma) provides two independent chronometers that help to assess the integrity of the parent-daughter systems and ages. Importantly, U-Pb dating of zircons can be applied to rocks from the Earth’s earliest history to rocks only a few hundreds of thousands of years old.

The development of *in situ* analytical techniques within individual grains enabled complex, multiple growth histories to be measured. This was initially made possible through the development of the Sensitive High Resolution Ion MicroProbe (or SHRIMP) [39] and subsequently expanded dramatically with the development of laser ablation inductively coupled plasma mass spectrometry (LA-ICP-MS) [40] instrumentation. Multi-grain analyses could be done quickly and it became possible to analyse large mixed populations of grains from sediments. Zircon is also a physically resilient mineral and survives weathering, erosion and deposition processes with the accumulated zircon populations in sediments providing important information of their original source rocks and their ages. Given sufficient numbers of analyses per sample it is possible to identify the relative proportions of the sources of the detrital zircons. This has many outcomes (*e*.*g*. [41]), including establishing the maximum age of deposition (defined by the youngest zircon dates) through to deciphering regional or tectonic-scale geologic histories. Crucially, the grouping of zircon dates–shown as peaks in probability plots–provide clear signatures that can be used to correlate (or just as importantly, exclude any correlation) sediments from diverse areas.

Although geologists have used detrital zircon U-Pb dating for several decades, this approach has only recently been used to establish provenance of ceramic and stone sources in the archaeological environment. In 2010, author R. Armstrong and M. Leclerc (unpublished) unequivocally established a local source for ceramic sherds in Vanuatu using zircons extracted from volcanic tuff horizons and small sherds. Temper sands were sourced in the Solomon Islands, southwest Pacific [9] and more recently, pottery has been sourced to local geological units in Colombia [12]. In another application Bevins *et al*. [11] used U-Pb zircon dating to constrain the provenance of a sandstone from Stonehenge in the UK.

One of the unsolved issues in studies of the Plain of Jars is the source of the carved rock jars and how these were transported to their present sites. As the vast majority of the jars are carved from sandstones or other sediments of similar mineralogical composition, it was clear that use of the U-Pb zircon ages in the manner described above, could help to match jars to their original sources or outcrops.

The quarry for Site 1 jars has been suggested to be Site 21 (Poukeng). In order to test this supposition we collected samples from both sites. With permission from the Lao Department of Heritage, a geological hammer was used to remove a small, 5 x 11 x 4 cm sample from a damaged jar, 1020102, at Site 1 (sample 15). Two samples were taken at Site 21; one from a cracked, but *in situ*, incomplete jar (sample 12) measuring 9 x 8 x 6 cm and a further piece of natural sandstone (sample 13), measuring 18 x 12 x 8 cm, from the same locale.

U-Pb ages were measured on zircon samples from Jar 1020102 from Site 1 and compared to zircons from a sandstone outcrop and an unfinished jar at Site 21. Zircons were separated at the ANU using standard heavy liquid and Franz magnetic techniques and were mounted in epoxy together with the relevant U-Pb standards and polished to approximately half the average thickness of the grains. Transmitted and reflective microphotographs plus cathodoluminescence images (S1 Fig) were used to select crack- and inclusion-free areas for analysis. U-Th-Pb analyses (~25 mm diameter) were done using Sensitive High Resolution Ion MicroProbe (SHRIMP) RG on randomly selected grains to ensure no bias was involved in characterising the zircon populations. This technique is commonly used as a geochronological tool for establishing the maximum age and the provenance of detrital zircons in sedimentary rocks. The SHRIMP data have been reduced in a manner similar to that described by Williams and Claesson [42], with all age calculations and statistical assessments done utilising the Excel Macros SQUID 2 [43] and Isoplot [44]. Pb/U ratios were corrected for instrumental inter-element fractionation using the ratios measured on the standard zircon Temora 2 (416.8 ± 1.3 Ma) [45]. Standard zircon SL13 (U = 238 ppm) was used as the reference value for U and Th concentrations in zircon [46]. Common Pb corrections were based on the measured ^204^Pb and the relevant common Pb compositions from the Stacey and Kramers model [47]. For the cumulative probability plots, ages that are <800 Ma are reported using the ^204^Pb corrected ^206^Pb/^238^U system because of the errors that are associated with low yields of ^204^Pb, ^207^Pb and ^208^Pb from relatively young zircons. All ages *older* than 800 Ma are reported using ^204^Pb corrected ^207^Pb/^206^Pb ratios. The ^207^Pb/^206^Pb ages that are >15% discordant are not plotted. The ages are plotted on cumulative probability plots (Fig 13) using Isoplot [44] and the age groups were calculated using the mixture modeling algorithm of Sambridge and Compston [48], also available in Isoplot.

## Results

### Radiocarbon dating

**Fig 9:**
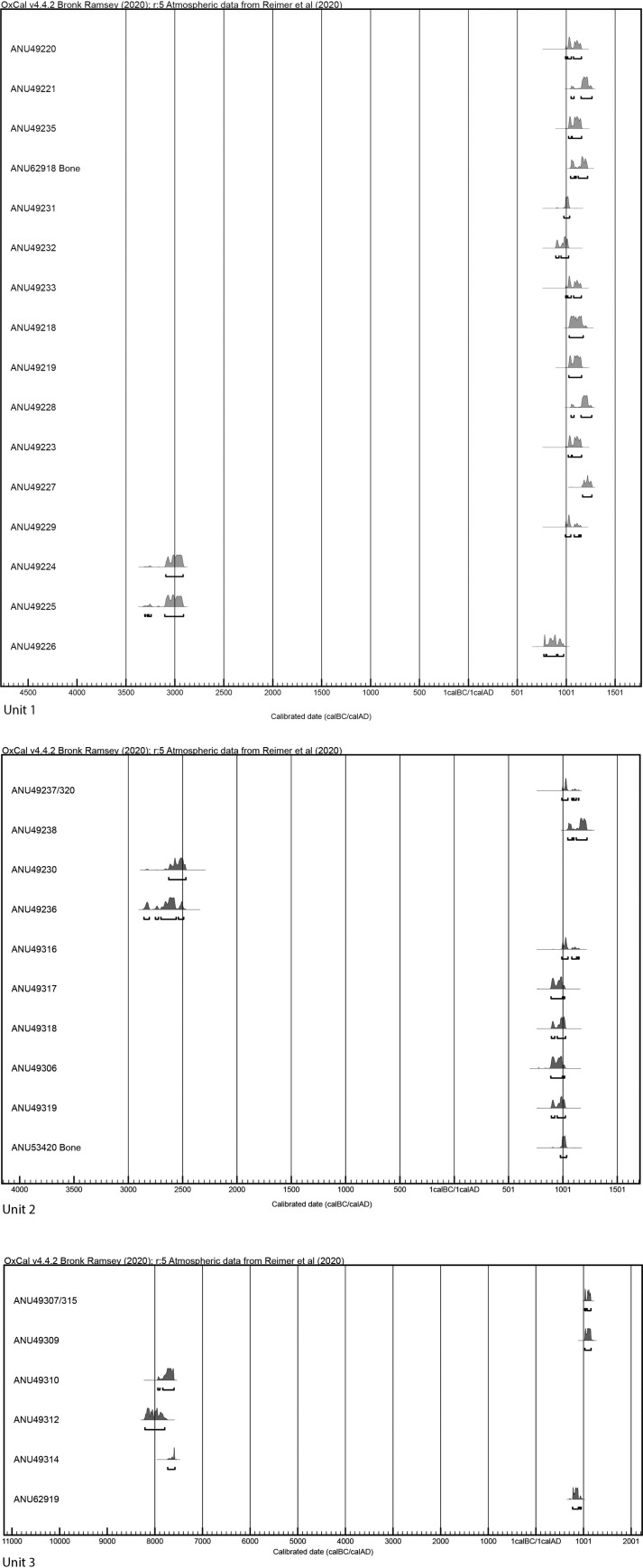
a) Calibrated 14C ages from Site 1, Unit 1 and b) Site 1, Units 2 and 3 using OxCal 4.4 and the IntCal 20 atmospheric calibration curve. The plot excludes ANU49313 (listed in S2 Table) as the date was anomalous.

Forty-six charcoal samples and two bone samples have been dated from Sites 1 and 2 (S2 Table and Table 1). The charcoal dates obtained from Site 1 reflect anthropogenic and/or natural activity from c. 8210–7794 calBC (ANU49312) to 1168–1264 calAD (ANU49227), with the majority of samples indicating that activity around the jars, including mortuary practice, occurred between c. the late ninth and thirteenth centuries AD (Fig 9A and 9B, S2 Table). The interpretation of wood charcoal dates sourced from burial contexts can be problematic [49], an example being in Unit 3 where dates obtained from bone (burial 6) were demonstrably more recent than the overlying matrix (S2 Table). However, the reliability of the dating here is demonstrated by results obtained directly from the human bone (ANU62918 and ANU62919, respectively, calAD 1046–1219 and calAD 772–950) that broadly support the charcoal dates (S2 Table). A possible explanation for the very early dates found high in the matrix in Unit 3 is that the excavation of pits for the placement of skeletal material and ceramic mortuary jars likely disturbed deeper contexts which was re-deposited higher in the stratigraphy.

**Table 1:** Dates of charcoal from three excavation units at Site 2.

Provenance	Lab number	Material	Depth below surface (m)	^14^C age BP	Calibrated age (Confidence 95.4%)
U1 1:2	ANU62924	Charcoal	0.18	929 ± 22	1035–1169 calAD
U1 1:1 F1	ANU62929	Charcoal	0.28	1142 ± 22	774–987 calAD
U1 1:2 F2	ANU62923	Charcoal	0.28	1063 ±23	897–1026 calAD
1:4 F5	ANU62930	Charcoal	0.35	150 ± 22	1667–1949 calAD*
U1 1:4	ANU62932	Charcoal	0.34	4456 ± 30	3337–3015 calBC
U1 1:4	ANU62926	Charcoal	0.34	137 ± 21	1675–1942 calAD*
U1 1:4 F5	ANU62920	Charcoal	0.45	8451 ± 32	7583–7483 calBC
U1 1:5 F3	ANU62921	Charcoal	0.91	948 ± 22	1033–1158 calAD
U1 1:5 F3	ANU62922	Charcoal	0.91	4277 ± 25	2921–2877 calBC
U2 1:1	ANU62931	Charcoal under jar W0018	0.11	2164 ± 25	355–105 calBC
U2 1:4	ANU62937	Charcoal	0.38	1332 ± 27	650–775 calAD
U2 1:5	ANU62933	Charcoal	0.53	1298 ± 26	662–774 calAD
U2 1:6	ANU62938	Charcoal	0.57	1279 ± 23	666–776 calAD
U3 1:3	ANU62925	Charcoal	0.25	1288 ± 32	659–820 calAD
U3 1:6	ANU62936	Charcoal	0.53	1308 ± 21	660–775 calAD
U3 1:6	ANU62935	Charcoal	0.51	1278 ± 23	666–777 calAD

**Fig 10:**
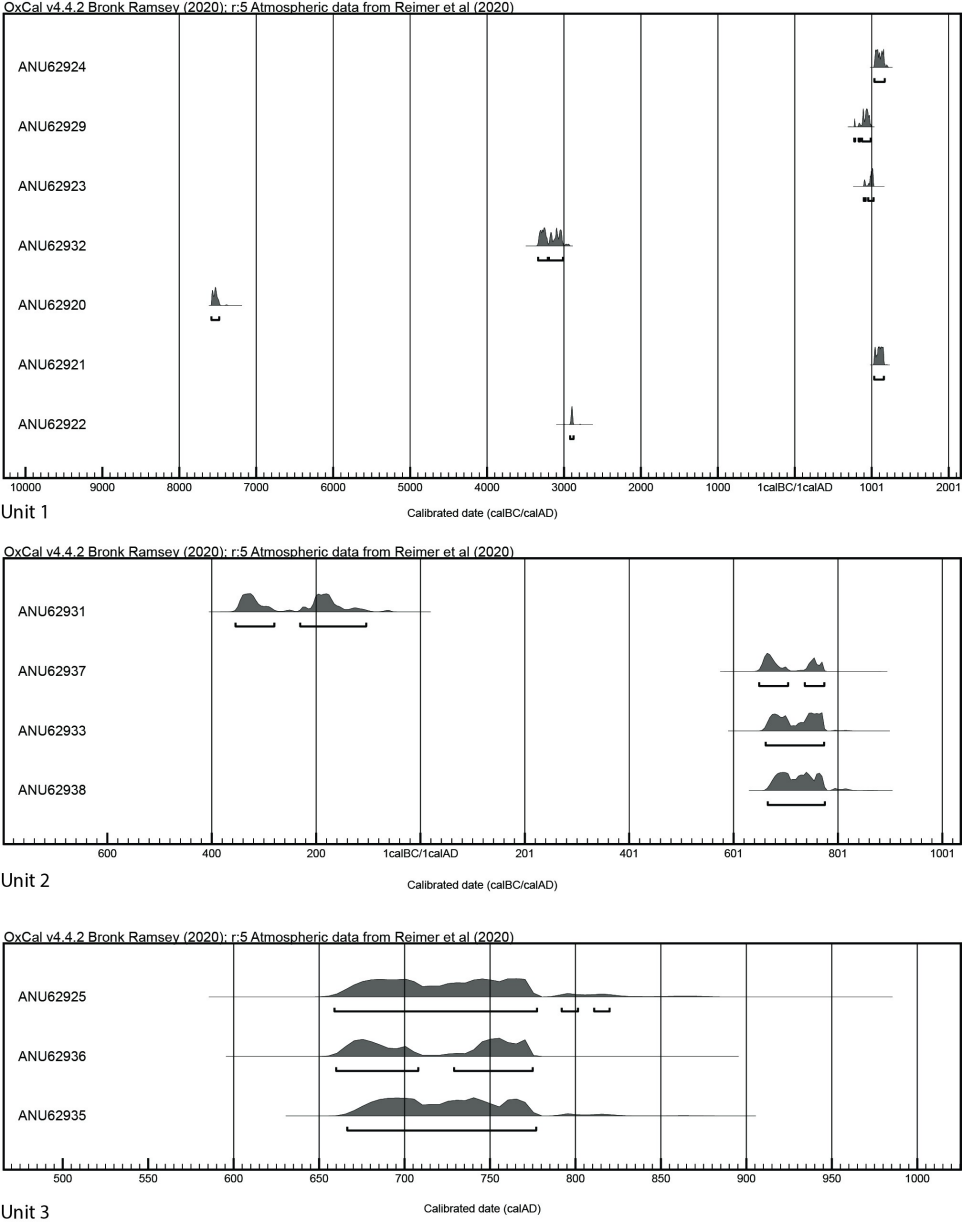
Calibrated 14C ages for charcoal from Site 2 (Units 1, 2, 3) using OxCal 4.4 and the IntCal 20 atmospheric calibration curve. The plot excludes ANU62930 and ANU62926 (listed in Table 1), as these modern-range results skewed plot presentation.

Calibrated radiocarbon ages are presented at 95.4% confidence, using OxCal v.4.4 and the IntCal 20 calibration curve [16,19,20]. Two results (ANU62930 and ANU62926), marked with an asterisk, calibrate into the modern era as well as being discordant with samples from a similar depth. These are presented here but are not included in Fig 10.

We also consider radiocarbon dates obtained from earlier research at Site 1 [15] that mirror the dates reported here, spanning a range between c. 7553–7051 calBC (ANU10764) to 1023–1214 calAD (ANU10767). Sayavongkhamdy took charcoal samples (ANU10764, ANU10765, ANU10766) from a burning layer c. 72–80 cm b.s. in an excavation in Group 2 at Site 1 which returned very early dates 7552–7083 calBC, 7553–7051 calBC and 7456–6829 calBC (S1 Table). Our excavations in 2016 recorded a similar burning layer at a depth of 78 cm b.s. from a sondage in Unit 1. A single charcoal sample from this sondage, however, returned a date of AD 772–975 (ANU49226) which is likely to be anomalous given that the dates obtained by Sayavongkhamdy correspond with charcoal dates obtained by the authors at a similar depth in Unit 3 (see ANU49314 in S2 Table).

The dates presented above pertain to activity around the megalithic jars. How this activity relates to the jars is unknown and a perennial problem has been the inability to date the placement of the megaliths themselves. A charcoal sample (ANU49227) excavated from beneath one of the jars at Site 1, Group 2 (Jar 01020061 located at N19.431023 E103.152399) returned a date of 1168–1264 calAD providing a possible *terminus post quem* for the jar above this context. It should be noted, however, that there was disturbance in the form of two secondary burials near the sample location. Bone from these secondary burials produced a similar date range to the sample from beneath the megalithic jar (see ANU 62918 in S2 Table).

Charcoal obtained from Site 2 rendered dates ranging from 7583–7483 calBC (ANU62920) to recent (Fig 10 and Table 1). A charcoal sample (ANU62931) taken from beneath the base of a damaged jar in the western group (W0018 located at N19.31877205 E103.15281324) returned a date of 355–105 calBC. Rather than relying solely on a single radiocarbon date to determine the timing of jar emplacement, support was sought from OSL dating of sediments beneath jars.

### OSL dating

OSL age estimates and associated data are summarised in Tables 2 and 3. The influence of jar material on the gamma dose rate to the OSL sample beneath the jar was assessed at Site 52 (sample GL17084). Using the fractional gamma D_r_ function in the R Luminescence package [36,37], the gamma D_r_ at the jar-sediment interface is 2.2% lower than that at 0.3 m depth beneath the jar, well within the bounds of uncertainty for gamma D_r_ associated with GL17084. Therefore, the radiochemistry, breadth and thickness of the jar’s base yields a relatively homogenous gamma field enveloping the OSL sample located directly beneath the jar.

**Table 2:** Variables and diagnostics underpinning OSL D_e_, D_r_ and Age data in Table 3.

Lab Code	Moisture content (%)	Depth (m)	Ge γ-spectrometry (*ex situ*)			α D_r_ (Gy.ka^-1^)	β D_r_ (Gy.ka^-1^)	γ D_r_ (Gy.ka^-1^)	Cosmic D_r_ (Gy.ka^-1^)	^226^Ra/^238^U	Preheat (°C for 10s)	Dose Recovery ratio	Low Dose Repeat Ratio	High Dose Repeat Ratio	Post-IR OSL Ratio
			K (%)	Th (ppm)	U (ppm)										
GL17083	8 ± 2	0.02	3.46 ± 0.28	18.87 ± 1.29	2.48 ± 0.31	0.75 ± 0.07	3.24 ± 0.27	1.84 ± 0.12	0.23 ± 0.05	0.85 ± 0.13	280	0.88 ± 0.02	0.95 ± 0.02	0.96 ± 0.03	1.02 ± 0.02
GL17084	5 ± 1	0.02	3.22 ± 0.28	14.96 ± 1.18	2.42 ± 0.33	0.67 ± 0.06	3.09 ± 0.25	1.67 ± 0.11	0.23 ± 0.05	0.56 ± 0.11	280	0.83 ± 0.02	0.95 ± 0.02	0.95 ± 0.03	1.01 ± 0.02
GL17084-Jar	5 ± 1	-0.02	3.04 ± 0.17	15.63 ± 0.81	2.24 ± 0.15	0.67 ± 0.05	2.95 ± 0.18	1.64 ± 0.08	-	0.74 ± 0.10	-	-	-	-	-
GL18100	23 ± 6	0.02	1.28 ±0.11	16.29 ± 0.99	2.67 ± 0.22	0.54 ±0.07	1.34 ±0.15	1.04 ±0.11	0.23 ±0.23	0.90 ± 0.12	240	1.01 ± 0.01	1.01 ±0.02	0.99 ±0.02	1.00 ±0.02
GL18101	23 ± 6	0.10	1.35 ±0.10	17.23 ± 0.94	2.66 ± 0.19	0.56 ±0.07	1.39 ±0.16	1.08 ±0.11	0.22 ±0.03	0.99 ± 0.12	240	0.99 ± 0.01	1.00 ±0.02	1.00 ±0.02	0.99 ±0.02
GL18102	23 ± 6	0.20	1.13 ±0.09	16.71 ± 0.85	3.04 ± 0.18	0.58 ±0.07	1.30 ±0.14	1.06 ±0.11	0.22 ±0.03	1.03 ± 0.11	240	0.99 ± 0.02	0.99 ±0.02	1.02 ±0.02	1.00 ±0.02
GL18103	21 ± 5	0.02	1.18 ±0.09	16.98 ± 0.90	2.82 ± 0.17	0.59 ±0.07	1.35 ±0.14	1.09 ±0.10	0.23 ±0.23	0.97 ± 0.10	220	0.99 ± 0.01	1.00 ±0.02	1.01 ±0.02	0.98 ±0.02
GL18104	21 ± 5	0.10	1.37 ±0.10	17.05 ± 0.91	2.93 ± 0.19	0.60 ±0.07	1.47 ±0.15	1.13 ±0.11	0.22 ±0.05	0.92 ± 0.11	240	1.03 ± 0.01	1.00 ±0.02	1.00 ±0.02	1.00 ±0.02
GL18105	21 ± 5	0.20	1.46 ±0.11	17.54 ± 0.97	3.18 ± 0.21	0.63 ±0.08	1.56 ±0.16	1.18 ±0.11	0.22 ±0.03	0.89 ± 0.11	240	1.00 ± 0.02	1.00 ±0.02	1.01 ±0.02	1.01 ±0.02
GL18106	22 ± 5	0.30	1.33 ±0.09	17.29 ± 0.89	3.01 ± 0.18	0.61 ±0.07	1.45 ±0.15	1.13 ±0.11	0.22 ±0.02	0.90 ± 0.10	240	0.99 ± 0.02	1.00 ±0.02	1.01 ±0.02	0.99 ±0.02

**Table 3:** OSL dates from multi-grain aliquots of natural 5–15 μm sedimentary quartz located beneath Jars 052020052 and 052030064 at Site 52, and Jars W0013 and W0021 at Site 2.

Jar No#	Sample Position below jar	Lab Code	D_r_ (Gy.ka^-1^)	D_e_ (Gy)	Age (ka)	Date
Jar 0064 (Site 52)	Directly under jar	GL17083	6.06 ± 0.30	261.8 ± 11.6	43.2 ± 2.9	44,110 BC– 38,310 BC
Jar 0052 (Site 52)	Directly under jar	GL17084	5.65 ± 0.29	242.6 ± 10.1	42.9 ± 2.8	43,720 BC– 38,100 BC
Jar W0013 (Site 2)	Directly under jar	GL18100	3.14 ± 0.30	9.6 ± 0.3	3.06 ± 0.31	1350 BC–730 BC
Jar W0013 (Site 2)	10cm below	GL18101	3.25 ± 0.21	9.2 ± 0.3	2.82 ± 0.21	1010 BC–590 BC
Jar W0013 (Site 2)	20cm below	GL18102	3.16 ± 0.19	16.7 ± 0.6	5.28 ± 0.38	3640 BC–2880 BC
Jar W0021 (Site 2)	Directly under jar	GL18103	3.26 ± 0.29	8.6 ± 0.3	2.62 ± 0.25	860 BC–350 BC
Jar W0021 (Site 2)	10cm below	GL18104	3.43 ± 0.21	11.0 ± 0.4	3.20 ± 0.22	1410 BC–960 BC
Jar W0021 (Site 2)	20cm below	GL18105	3.59 ± 0.22	19.6 ± 0.7	5.47 ± 0.38	3840 BC–3070 BC
Jar W0021 (Site 2)	30cm below	GL18106	3.41 ± 0.20	31.7 ± 1.1	9.31 ± 0.65	7930 BC–6640 BC

Age estimates are expressed relative to the year of sampling (2017–2019). Uncertainties in age are quoted at 1σ confidence and reflect combined systematic and experimental variability. See Table 2 for further details.

The jar bases could not be sampled at Site 2, so the OSL age estimates of these samples are based on lithogenic and cosmogenic D_r_ alone. Assuming the radiochemistry of the sandstone comprising the jars is similar between Sites 2 and 52, the assessment of total D_r_ should be considered a minimum value and the corresponding OSL age a maximum value.

The source of the fine quartz silt beneath the jars at Sites 2 and 52 was probably wind and rain-borne dust. This is consistent with the slow accumulation rate at Site 2 (<0.05 mm/yr) demonstrated by the difference in age between the samples from 20 cm and 30 cm depth (5.3–5.5 ka and 9.3 ka respectively; Table 3). Because the silt is probably wind-blown, overestimation of OSL ages as a result of partial resetting of the OSL signal prior to burial is unlikely.

At Site 52 the OSL ages from beneath the two jars are coeval (~43 ka), even though the reliability of the age estimates is compromised by poor dose recovery ratios (significantly less than unity) and, for sample GL17084, significant U disequilibrium. These ages are far in excess of those previously reported for human activity associated with jar sites; the jars were likely worked with metal tools that were not in use in mainland Southeast Asia until the second millennium BC [50,51]. The c. 40 ka ages at Site 52 probably date primary deposition of wind-blown silt.

**Fig 11:**
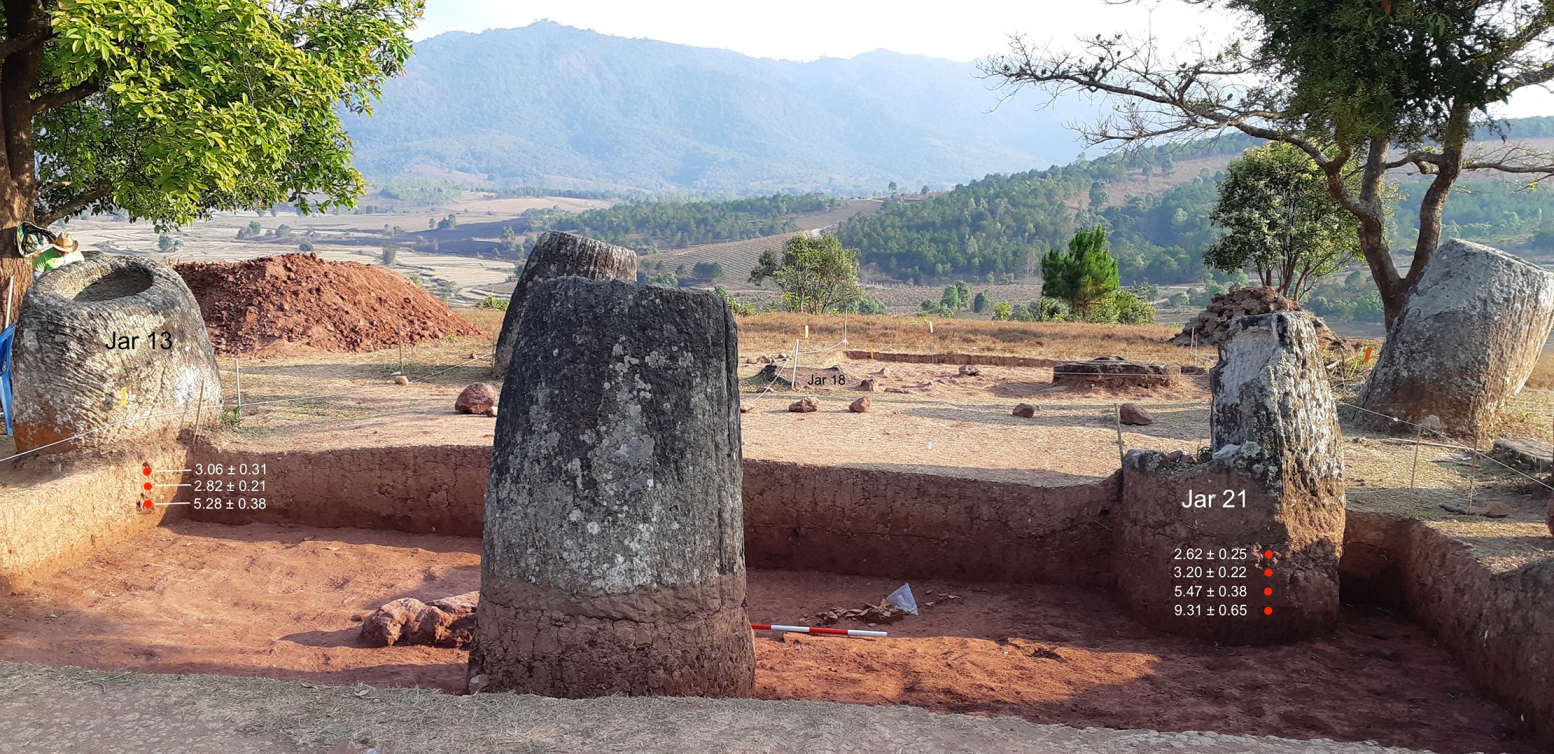
Location of OSL samples taken from beneath Jars W0013 and W0021 at Site 2. The remains of Jar W0018 can be seen in the background. Figures are reported as kya.

At Site 2, the consistent age of the sediment at 20 cm depth beneath the two jars 6.3 m apart (5.3–5.5 ka; Fig 11) demonstrates that the stratigraphy at this depth has not been significantly disturbed, suggesting that any pedoturbation is confined to shallower depths.

The four OSL dates from the upper 10 cm of sediment beneath the two jars at Site 2 (Fig 11) are statistically indistinguishable, i.e. they all overlap within 2σ (Table 3; note that they are statistically separate from the underlying dates). Averaging these four dates using the nonparametric method of Rock et al. [52] to estimate 87.5% confidence intervals around the median gives an age range of 2.7–3.3 ka (1240 BC to 660 BC expressed relative to 2019, the year of sampling). The consistency of the ages in the upper 10 cm implies that this layer of soil was disturbed before jar emplacement, and that this stopped when the jars were in place. The disturbance could have been caused by pedoturbation of the upper soil, or the upper 10 cm of the soil could have been dug over when the jars were emplaced. In either case, location of the jars at Site 2 probably occurred between 1240 BC and 660 BC.

### Zircon geochronology

**Fig 12:**
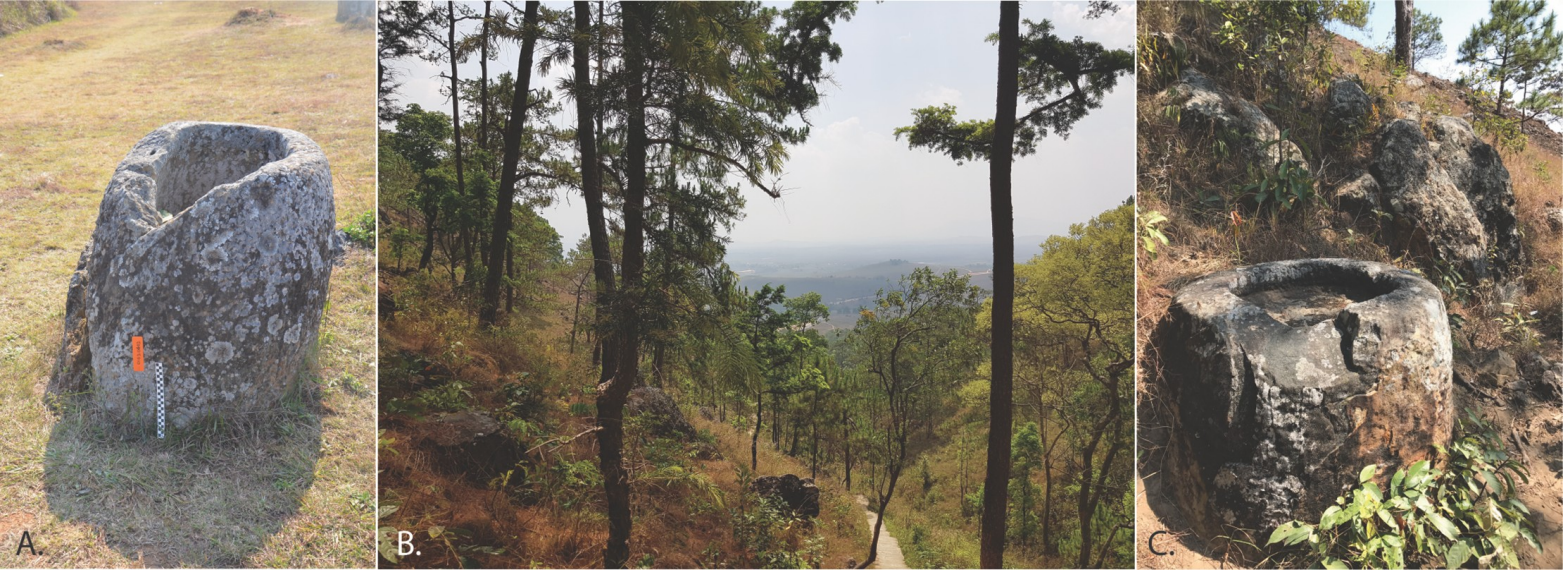
**A**. Jar 102 from Site 1, **B**. Site 21 (Phoukeng Quarry), photo courtesy Simon Tener. **C**. Partially completed jar at Phoukeng Quarry.

**Fig 13:**
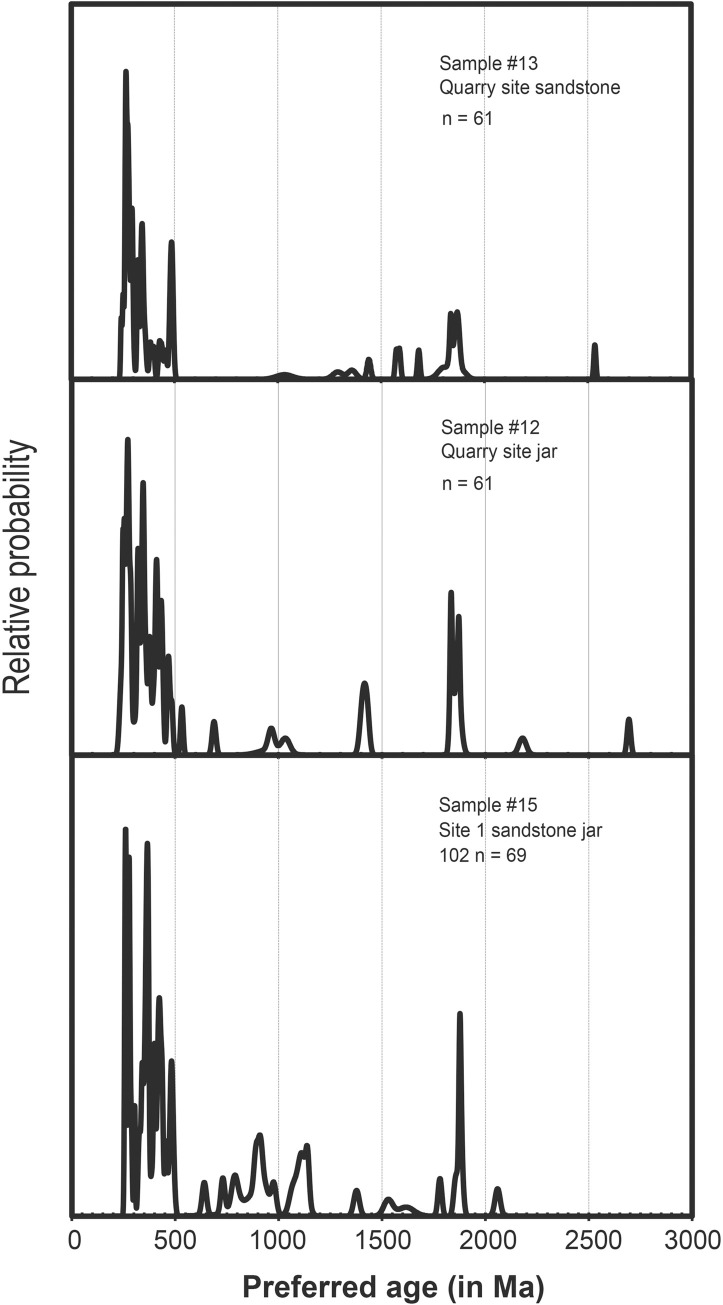
Stacked zircon U-Pb age cumulative probability curves for three samples from Sites 1 and 21. Samples 12 (an uncompleted jar) and 13 (a nearby sandstone outcrop) are from the quarry site and sample 15 is jar 01020125 from Site 1. n = the number of analyses done per sample.

Jar samples from Site 1 were collected to compare with potential source material (an unfinished jar and sandstone outcrop) from Site 21, which is c. 8 km to the northwest of Site 1 and is the closest quarry to the site (Fig 12, and Tables 4–6). Zircons were separated from these samples and the U-Pb ages measured on representative populations using a SHRIMP. A total of 61 different zircon grains were analysed in each of the samples #12 and #13, and a total of 69 grains in sample #15. To facilitate visual comparison between the zircon U-Pb ages the results are plotted as stacked cumulative probability plots in Fig 13. The dominant age bracket for all three samples is between 260 to 500 Ma with the lower date providing a *maximum* age of deposition of the sandstones. Using the mixture modeling algorithm in Isoplot [44] it is possible to deconvolute the dates from each sample and identify and quantify the different age components for each sample. These data are listed in S3 Table and identify 5 groups within each sample and their uncertainties. It is clear that all three samples have almost identical age groupings between ~260 and 500 Ma. In order of increasing age the groups are 268–270 Ma, 335–336 Ma, 364–390 Ma, 411–439 Ma and 471–485 Ma. Older grains are present in minor numbers and are not statistically significant apart from a sub-population between 1836 and 1876 Ma.

**Table 4:** SHRIMP U-Th-Pb data for sample 12. Uncertainties in the ratios and ages are reported at 1σ.

Grain spot	% ^206^Pb_c_	ppm U	ppm Th	^232^Th/^238^U	±%	(1) ppm ^206^Pb*	(1) ^206^Pb/^238^U Age		(1) ^207^Pb/^206^Pb Age		% Dis- cor- dant	(1) ^207^Pb*^/206^Pb*	±%	(1) ^207^Pb*^/235^U	±%	(1) ^206^Pb*^/238^U	±%	err corr
1.1	0.00	202	126	0.65	0.44	8	288	±4	277	±50	-4	0.0502	3.1	0.32	3.4	0.046	1.5	0.4
2.1	0.19	127	56	0.45	0.54	5	270	±4	170	±100	-60	0.0452	3.5	0.26	3.8	0.043	1.6	0.4
3.1	0.09	503	304	0.62	0.36	30	433	±5	403	±29	-8	0.0538	1.7	0.51	2.0	0.069	1.4	0.5
4.1	0.18	304	183	0.62	0.41	20	465	±6	455	±41	-2	0.0494	2.2	0.51	2.5	0.074	1.5	0.4
5.1	0.23	171	144	0.87	0.44	8	347	±5	304	±75	-15	0.0519	4.7	0.40	5.2	0.055	1.6	0.4
6.1	0.02	329	59	0.18	0.51	97	1895	±21	1873	±9	-1	0.1146	0.5	5.40	1.4	0.342	1.3	0.9
7.1	0.76	64	19	0.30	0.80	2	259	±4	163	±229	-60	0.0597	3.6	0.34	3.9	0.042	1.7	0.4
8.1	0.16	448	186	0.43	0.39	17	275	±3	205	±49	-35	0.0506	1.9	0.30	2.1	0.044	1.4	0.5
9.1	0.11	330	131	0.41	0.42	68	1389	±16	1402	±13	+1	0.0888	0.8	2.94	1.4	0.240	1.4	0.8
10.1	0.10	363	292	0.83	0.38	18	357	±4	324	±42	-10	0.0512	3.1	0.40	3.4	0.057	1.5	0.4
11.1	0.19	96	73	0.79	0.52	5	343	±5	370	±90	+7	0.0580	4.5	0.44	5.0	0.055	1.8	0.4
12.1	0.11	241	230	0.99	0.40	36	1019	±13	1034	±21	+2	0.0728	2.2	1.72	2.7	0.171	1.6	0.5
13.1	0.13	263	132	0.52	0.43	13	365	±6	362	±49	-1	0.0542	2.2	0.44	2.5	0.058	1.8	0.5
14.1	0.15	343	127	0.38	0.43	13	270	±5	249	±55	-9	0.0514	2.2	0.30	2.5	0.043	1.8	0.5
15.1	0.10	244	90	0.38	0.46	9	271	±4	200	±60	-36	0.0494	2.3	0.29	2.6	0.043	1.4	0.4
16.1	0.06	251	130	0.54	0.43	14	414	±5	370	±42	-12	0.0527	2.1	0.48	2.4	0.066	1.5	0.5
17.1	0.00	242	117	0.50	0.44	15	440	±6	443	±34	+1	0.0549	2.0	0.53	2.2	0.071	1.4	0.5
18.1	0.00	241	88	0.38	0.46	9	265	±3	232	±48	-14	0.0498	2.5	0.29	2.8	0.042	1.5	0.5
19.1	0.07	259	139	0.55	0.43	55	1420	±17	1416	±14	-0	0.0922	1.0	3.14	1.5	0.247	1.4	0.8
20.1	0.49	148	120	0.84	0.44	7	330	±6	280	±103	-18	0.0540	4.5	0.39	5.1	0.053	2.3	0.4
21.1	0.05	376	187	0.51	0.38	80	1427	±16	1430	±11	+0	0.0898	0.8	3.07	1.4	0.248	1.4	0.8
22.1	0.56	64	33	0.53	0.65	3	341	±5	377	±156	+10	0.0507	4.3	0.38	4.7	0.054	1.8	0.4
23.1	0.06	411	183	0.46	0.39	14	254	±3	258	±43	+1	0.0528	1.9	0.29	2.1	0.040	1.4	0.5
24.1	0.24	265	93	0.36	0.44	10	283	±4	269	±65	-5	0.0522	2.1	0.32	2.3	0.045	1.4	0.5
25.1	0.03	495	309	0.65	0.35	136	1794	±20	1831	±7	+2	0.1120	0.8	4.96	1.3	0.321	1.4	0.8
26.1	--	248	143	0.60	0.41	17	484	±6	477	±30	-1	0.0575	1.9	0.62	2.2	0.078	1.4	0.5
27.1	0.00	1179	1257	1.10	0.32	351	1918	±20	1872	±5	-3	0.1114	0.8	5.30	1.3	0.345	1.4	0.8
28.1	0.06	280	414	1.53	0.35	12	320	±4	383	±42	+17	0.0482	16.7	0.34	17.4	0.051	1.8	0.4
29.1	0.58	2634	734	0.29	0.32	89	247	±3	251	±37	+1	0.0487	1.3	0.26	1.7	0.039	1.3	0.6
30.1	0.11	649	577	0.92	0.33	42	470	±6	469	±24	-0	0.0561	2.2	0.59	2.5	0.076	1.5	0.5
31.1	0.00	140	64	0.47	0.49	5	279	±4	286	±57	+3	0.0500	2.9	0.30	3.2	0.044	1.5	0.4
32.1	0.05	249	130	0.54	0.40	15	430	±5	403	±36	-7	0.0551	1.9	0.52	2.1	0.069	1.4	0.5
33.1	0.08	47	21	0.47	0.76	16	2190	±32	2180	±18	-1	0.1338	1.3	7.45	1.9	0.404	1.8	0.8
34.1	0.02	298	56	0.20	0.49	83	1806	±20	1860	±9	+3	0.1144	0.5	5.11	1.3	0.324	1.3	0.9
35.1	0.20	126	52	0.43	0.53	12	688	±9	671	±47	-3	0.0659	1.8	1.03	2.1	0.113	1.5	0.5
36.1	0.30	46	22	0.49	0.73	2	395	±7	372	±128	-6	0.0543	3.9	0.47	4.3	0.063	1.9	0.4
37.1	0.34	136	104	0.79	0.44	6	347	±7	286	±87	-22	0.0569	3.7	0.44	4.2	0.056	2.4	0.5
38.1	0.03	2808	1905	0.70	0.30	157	407	±5	407	±22	+0	0.0553	1.3	0.50	1.7	0.065	1.3	0.6
39.1	0.07	214	106	0.51	0.43	58	1753	±20	1850	±11	+6	0.1131	0.8	4.87	1.4	0.312	1.4	0.8
40.1	0.29	115	52	0.46	0.53	5	347	±5	318	±91	-9	0.0536	2.8	0.41	3.0	0.055	1.6	0.4
41.1	0.20	287	291	1.05	0.36	15	381	±5	406	±46	+6	0.0546	4.7	0.46	5.1	0.061	1.6	0.4
42.1	0.04	295	134	0.47	0.41	59	1348	±21	1415	±12	+5	0.0895	1.0	2.87	1.8	0.233	1.8	0.9
43.1	0.25	74	37	0.52	0.61	3	320	±5	292	±221	-10	0.0531	8.7	0.37	9.0	0.051	1.7	0.3
44.1	0.21	722	41	0.06	0.53	97	934	±11	965	±15	+3	0.0723	0.6	1.56	1.3	0.156	1.2	0.9
45.1	0.13	503	148	0.30	0.76	37	533	±6	591	±25	+10	0.0592	1.3	0.70	1.6	0.086	1.3	0.6
46.1	0.10	534	585	1.13	0.97	30	411	±5	414	±28	+1	0.0536	7.4	0.49	8.0	0.066	1.6	0.5
47.1	0.01	1582	105	0.07	3.90	443	1818	±22	1834	±4	+1	0.1124	0.5	5.05	1.5	0.326	1.4	0.9
48.1	0.32	456	335	0.76	0.35	23	375	±5	415	±42	+10	0.0544	2.1	0.45	2.4	0.060	1.4	0.5
49.1	0.03	741	33	0.05	1.39	207	1813	±19	1839	±6	+2	0.1126	0.4	5.04	1.3	0.325	1.2	1.0
50.1	--	417	53	0.13	0.50	118	1830	±20	1880	±13	+3	0.1143	0.8	5.17	1.5	0.328	1.3	0.8
51.1	0.49	66	30	0.47	0.57	3	319	±12	361	±139	+12	0.0540	4.5	0.38	5.2	0.051	4.1	0.6
52.1	0.00	159	55	0.36	0.39	5	251	±9	186	±56	-36	0.0538	3.8	0.30	4.4	0.040	3.9	0.6
53.1	0.04	1066	184	0.18	0.45	60	414	±14	392	±18	-6	0.0545	1.0	0.50	3.5	0.066	3.5	1.0
54.1	0.33	64	71	1.13	0.42	2	254	±9	235	±149	-9	0.0542	14.2	0.30	15.7	0.040	4.6	0.5
55.1	0.45	495	197	0.41	0.56	16	235	±8	253	±61	+7	0.0507	2.4	0.26	3.7	0.037	3.6	0.8
56.1	0.25	99	98	1.03	0.35	6	420	±16	447	±76	+6	0.0567	6.7	0.53	8.1	0.067	4.7	0.6
57.1	0.00	38	12	0.32	0.85	2	294	±11	226	±103	-31	0.0512	5.2	0.33	5.9	0.047	4.1	0.5
58.1	0.06	122	106	0.90	0.32	50	2563	±79	2695	±9	+6	0.1846	1.2	12.42	3.5	0.488	4.1	1.0
59.1	0.27	318	271	0.88	0.34	14	326	±11	368	±54	+12	0.0571	3.5	0.41	4.6	0.052	3.9	0.7
60.1	--	451	168	0.38	0.23	15	247	±8	249	±58	+1	0.0487	3.1	0.26	4.2	0.039	3.5	0.7
61.1	0.06	325	81	0.26	0.32	43	922	±33	962	±55	+4	0.0707	2.8	1.50	4.7	0.154	4.0	0.8

Errors are 1-sigma; Pbc and Pb* indicate the common and radiogenic portions, respectively. Error in Standard calibration was 0.30% (not included in above errors but required when comparing data from different mounts). (1) Common Pb corrected using measured 204Pb.

**Table 5:** SHRIMP U-Th-Pb data for sample 13. Uncertainties in the ratios and ages are reported at 1σ.

Grain spot	% ^206^Pb_c_	ppm U	ppm Th	^232^Th/^238^U	±%	(1) ppm ^206^Pb*	(1) ^206^Pb/^238^U Age		(1) ^207^Pb/^206^Pb Age		% Dis- cor- dant	(1) ^207^Pb*^/206^Pb*	±%	(1) ^207^Pb*^/235^U	±%	(1) ^206^Pb*^/238^U	±%	err corr
1.1	--	293	78	0.28	0.47	86	1897	±22	1869	±9	-2	0.1143	0.5	5.39	1.4	0.342	1.3	0.94
2.1	0.04	564	350	0.64	0.36	22	281	±4	269	±34	-5	0.0516	1.5	0.32	2.0	0.045	1.3	0.66
3.1	0.00	96	36	0.39	0.63	4	296	±4	271	±71	-9	0.0517	3.1	0.33	3.4	0.047	1.5	0.45
4.1	0.00	78	64	0.86	0.55	15	1321	±26	1290	±25	-3	0.0839	1.3	2.63	2.5	0.227	2.2	0.87
5.1	0.11	515	258	0.52	0.37	34	483	±6	459	±29	-5	0.0562	1.3	0.60	1.8	0.078	1.3	0.71
6.1	0.12	208	130	0.65	0.43	7	265	±4	248	±67	-7	0.0512	2.9	0.30	3.2	0.042	1.4	0.43
7.1	--	221	111	0.52	0.45	8	282	±4	318	±48	+12	0.0527	2.1	0.33	2.5	0.045	1.4	0.55
8.1	0.14	166	46	0.29	0.56	7	291	±4	288	±73	-1	0.0521	3.2	0.33	3.5	0.046	1.4	0.41
9.1	0.02	672	684	1.05	0.34	45	483	±6	460	±20	-5	0.0562	0.9	0.60	1.6	0.078	1.3	0.81
10.1	0.42	113	176	1.60	0.44	4	265	±4	267	±128	+1	0.0516	5.6	0.30	5.8	0.042	1.6	0.27
11.1	0.09	280	212	0.78	0.39	10	264	±3	230	±55	-15	0.0508	2.4	0.29	2.7	0.042	1.4	0.50
12.1	0.09	402	196	0.50	0.39	21	383	±5	385	±37	+0	0.0543	1.6	0.46	2.1	0.061	1.3	0.63
13.1	0.51	70	52	0.76	0.58	3	356	±6	234	±153	-54	0.0509	6.6	0.40	6.8	0.057	1.7	0.24
14.1	--	64	33	0.54	0.66	3	355	±11	406	±75	+13	0.0549	3.4	0.43	4.7	0.057	3.3	0.70
15.1	0.00	62	33	0.55	0.67	3	341	±6	391	±78	+13	0.0545	3.5	0.41	3.9	0.054	1.7	0.43
16.1	0.11	217	75	0.36	0.48	9	300	±6	250	±62	-21	0.0512	2.7	0.34	3.3	0.048	1.9	0.57
17.1	0.13	358	152	0.44	0.40	14	292	±4	276	±50	-6	0.0518	2.2	0.33	2.6	0.046	1.3	0.52
18.1	0.13	178	76	0.44	0.48	7	276	±4	292	±70	+6	0.0522	3.1	0.31	3.4	0.044	1.4	0.42
19.1	0.13	199	109	0.56	0.45	7	269	±4	238	±71	-14	0.0509	3.1	0.30	3.4	0.043	1.4	0.41
20.1	0.07	202	171	0.88	0.41	13	456	±6	423	±44	-8	0.0553	2.0	0.56	2.4	0.073	1.4	0.58
21.1	0.09	170	76	0.46	0.49	9	399	±5	374	±53	-7	0.0541	2.3	0.48	2.7	0.064	1.4	0.52
22.1	0.10	260	103	0.41	0.45	18	490	±6	468	±39	-5	0.0564	1.7	0.61	2.2	0.079	1.4	0.62
23.1	0.26	153	68	0.46	0.51	7	346	±5	311	±83	-11	0.0526	3.7	0.40	3.9	0.055	1.4	0.37
24.1	0.02	689	250	0.37	0.38	40	427	±5	416	±37	-3	0.0551	1.6	0.52	2.1	0.068	1.3	0.62
25.1	0.31	158	84	0.55	0.48	6	262	±4	296	±97	+12	0.0522	4.3	0.30	4.5	0.041	1.4	0.32
26.1	0.05	320	87	0.28	0.46	90	1835	±21	1857	±9	+1	0.1136	0.5	5.16	1.4	0.329	1.3	0.94
27.1	0.08	293	46	0.16	0.54	81	1806	±26	1865	±9	+4	0.1141	0.5	5.08	1.7	0.323	1.6	0.95
28.1	0.15	126	108	0.88	0.47	6	345	±5	351	±77	+2	0.0535	3.4	0.41	3.7	0.055	1.6	0.42
29.1	0.05	306	43	0.15	0.55	88	1866	±22	1876	±9	+1	0.1147	0.5	5.31	1.4	0.336	1.3	0.94
30.1	0.02	1039	294	0.29	0.36	35	251	±3	235	±25	-7	0.0509	1.1	0.28	1.7	0.040	1.3	0.76
31.1	0.03	482	267	0.57	0.37	178	2306	±25	2535	±5	+11	0.1677	0.3	9.94	1.3	0.430	1.3	0.97
32.1	1.49	30	16	0.57	0.88	7	1526	±28	2240	±49	+36	0.1410	2.8	5.20	3.5	0.267	2.0	0.58
33.1	0.31	174	63	0.37	0.51	45	1683	±30	1802	±28	+8	0.1102	1.6	4.53	2.6	0.298	2.0	0.79
34.1	0.13	352	296	0.87	0.38	13	273	±4	270	±50	-1	0.0516	2.2	0.31	2.5	0.043	1.3	0.53
35.1	0.01	919	535	0.60	0.34	216	1560	±18	1574	±6	+1	0.0973	0.3	3.68	1.3	0.274	1.3	0.97
36.1	0.09	318	341	1.11	0.37	19	438	±6	422	±37	-4	0.0552	1.7	0.54	2.1	0.070	1.3	0.63
37.1	0.13	399	126	0.33	0.43	109	1778	±21	1837	±9	+4	0.1123	0.5	4.92	1.4	0.318	1.3	0.94
38.1	0.19	92	87	0.97	0.51	13	1000	±14	1032	±39	+3	0.0736	1.9	1.70	2.5	0.168	1.6	0.62
39.1	--	354	137	0.40	0.80	13	266	±4	237	±49	-12	0.0509	2.1	0.30	2.5	0.042	1.3	0.53
40.1	--	85	45	0.55	0.60	6	490	±7	507	±71	+4	0.0574	3.2	0.62	3.6	0.079	1.6	0.44
41.1	0.09	34	23	0.70	0.82	9	1685	±56	1809	±30	+8	0.1106	1.7	4.55	4.1	0.299	3.8	0.91
42.1	0.11	383	161	0.44	0.41	71	1255	±20	1359	±20	+8	0.0869	1.0	2.58	2.0	0.215	1.7	0.86
43.1	0.26	618	79	0.13	0.47	27	322	±4	330	±42	+2	0.0530	1.9	0.37	2.3	0.051	1.3	0.58
44.1	0.02	1395	60	0.04	0.50	351	1655	±19	1837	±5	+11	0.1123	0.3	4.53	1.3	0.293	1.3	0.98
45.1	0.17	128	143	1.16	0.45	6	323	±5	373	±81	+14	0.0541	3.6	0.38	3.9	0.051	1.5	0.38
46.1	0.12	602	289	0.50	2.04	22	272	±3	258	±39	-6	0.0514	1.7	0.31	2.2	0.043	1.3	0.61
47.1	0.15	137	66	0.50	0.52	6	323	±5	239	±79	-36	0.0510	3.4	0.36	3.7	0.051	1.5	0.39
48.1	0.32	73	37	0.53	0.63	3	276	±5	276	±142	-0	0.0518	6.2	0.31	6.4	0.044	1.7	0.27
49.1	0.05	538	182	0.35	0.39	18	242	±3	210	±39	-16	0.0503	1.7	0.27	2.1	0.038	1.3	0.61
50.1	0.00	144	109	0.78	0.46	5	265	±4	370	±103	+29	0.0540	4.6	0.31	4.8	0.042	1.5	0.32
51.1	0.01	706	60	0.09	0.35	178	1690	±19	1682	±6	-1	0.1032	0.3	4.26	1.3	0.300	1.2	1.0
52.1	0.13	240	285	1.23	0.69	16	489	±5	457	±38	-7	0.0561	1.7	0.61	2.1	0.079	1.1	0.5
53.1	0.00	813	306	0.39	1.24	30	277	±4	258	±23	-8	0.0514	1.0	0.31	1.8	0.044	1.4	0.8
54.1	0.00	77	78	1.05	0.40	4	342	±5	310	±68	-11	0.0526	3.0	0.39	3.3	0.054	1.4	0.4
55.1	0.12	191	65	0.35	0.36	6	254	±6	263	±66	+3	0.0515	2.9	0.29	3.6	0.040	2.2	0.6
56.1	0.01	435	146	0.35	0.24	92	1448	±17	1441	±9	-1	0.0907	0.5	3.15	1.4	0.252	1.3	0.9
57.1	--	133	65	0.51	0.38	6	336	±4	351	±65	+4	0.0535	2.9	0.40	3.1	0.054	1.2	0.4
58.1	0.01	807	453	0.58	0.15	191	1599	±18	1589	±6	-1	0.0981	0.3	3.81	1.3	0.282	1.3	1.0
59.1	0.01	393	44	0.12	0.42	114	1908	±24	1892	±22	-1	0.1158	1.2	5.50	1.9	0.344	1.5	0.8
60.1	--	607	413	0.70	0.16	24	296	±3	289	±46	-3	0.0521	2.0	0.34	2.3	0.047	1.1	0.5
61.1	0.00	105	88	0.86	0.36	7	478	±6	468	±45	-2	0.0564	2.0	0.60	2.4	0.077	1.3	0.5

Errors are 1-sigma; Pbc and Pb* indicate the common and radiogenic portions, respectively. Error in Standard calibration was 0.30% (not included in above errors but required when comparing data from different mounts). (1) Common Pb corrected using measured 204Pb.

**Table 6:** SHRIMP U-Th-Pb data for sample 15. Uncertainties in the ratios and ages are reported at 1σ.

Grain spot	% ^206^Pb_c_	ppm U	ppm Th	^232^Th/^238^U	±%	(1) ppm ^206^Pb*	(1) ^206^Pb/^238^U Age		(1) ^207^Pb/^206^Pb Age		% Dis- cor- dant	(1) ^207^Pb*^/206^Pb*	±%	(1) ^207^Pb*^/235^U	±%	(1) ^206^Pb*^/238^U	±%	err corr
1.1	0.06	501	375	0.77	0.20	21	303	±3	305	±28	+1	0.0525	1.2	0.35	1.6	0.048	1.1	0.6
2.1	0.06	183	103	0.58	0.82	9	352	±7	352	±42	-0	0.0535	1.9	0.41	2.8	0.056	2.1	0.7
3.1	0.06	168	61	0.37	0.36	49	1888	±18	1881	±10	-0	0.1151	0.6	5.40	1.2	0.340	1.1	0.9
4.1	0.03	604	132	0.22	0.91	153	1667	±24	1780	±9	+7	0.1088	0.5	4.43	1.7	0.295	1.6	1.0
5.1	0.06	786	46	0.06	0.37	70	640	±11	673	±13	+5	0.0620	0.6	0.89	1.8	0.104	1.7	0.9
6.1	0.99	662	359	0.56	0.50	24	263	±4	352	±117	+26	0.0535	5.2	0.31	5.5	0.042	1.7	0.3
7.1	0.00	190	134	0.73	1.01	31	1130	±15	1112	±15	-2	0.0766	0.7	2.02	1.6	0.192	1.4	0.9
8.1	0.03	371	74	0.21	0.30	108	1877	±26	1880	±11	+0	0.1150	0.6	5.36	1.7	0.338	1.6	0.9
9.1	0.14	694	356	0.53	0.18	26	275	±3	240	±34	-15	0.0510	1.5	0.31	1.8	0.044	1.0	0.6
10.1	0.20	177	93	0.55	0.33	11	455	±5	373	±54	-23	0.0540	2.4	0.55	2.7	0.073	1.2	0.4
11.1	0.15	3041	3652	1.24	0.37	108	262	±3	290	±14	+10	0.0521	0.6	0.30	1.5	0.042	1.4	0.9
12.1	0.08	473	310	0.68	0.18	28	436	±9	419	±23	-4	0.0552	1.0	0.53	2.3	0.070	2.0	0.9
13.1	0.29	161	106	0.68	0.28	9	424	±5	400	±56	-6	0.0547	2.5	0.51	2.8	0.068	1.3	0.5
14.1	0.03	866	64	0.08	0.94	108	874	±11	890	±10	+2	0.0687	0.5	1.38	1.4	0.145	1.3	0.9
15.1	0.10	117	61	0.54	0.36	30	1694	±45	2375	±10	+33	0.1525	0.6	6.32	3.1	0.300	3.0	1.0
16.1	0.11	283	152	0.56	0.24	11	276	±3	288	±43	+4	0.0521	1.9	0.31	2.3	0.044	1.3	0.6
17.1	0.00	690	38	0.06	0.40	204	1905	±22	1876	±4	-2	0.1147	0.2	5.44	1.4	0.344	1.4	1.0
18.1	--	193	32	0.17	0.75	26	940	±45	883	±49	-7	0.0685	2.4	1.48	5.7	0.157	5.2	0.9
19.1	0.00	268	245	0.95	1.10	13	361	±6	338	±30	-7	0.0532	1.3	0.42	2.3	0.058	1.8	0.8
20.1	--	74	29	0.40	0.50	4	429	±6	434	±50	+1	0.0555	2.2	0.53	2.7	0.069	1.6	0.6
21.1	0.00	474	348	0.76	0.17	138	1881	±32	1856	±10	-2	0.1135	0.5	5.30	2.0	0.339	2.0	1.0
22.1	--	51	25	0.51	0.54	3	361	±5	438	±65	+18	0.0556	2.9	0.44	3.2	0.058	1.3	0.4
23.1	0.15	175	179	1.06	0.24	27	1064	±19	1129	±19	+6	0.0773	1.0	1.91	2.2	0.180	2.0	0.9
24.1	0.03	993	191	0.20	5.08	57	420	±7	434	±15	+4	0.0556	0.7	0.52	1.9	0.067	1.7	0.9
25.1	0.19	195	182	0.96	1.68	9	334	±7	302	±55	-11	0.0524	2.4	0.38	3.2	0.053	2.1	0.7
26.1	0.11	88	46	0.54	0.42	5	418	±8	285	±65	-48	0.0520	2.8	0.48	3.4	0.067	2.0	0.6
27.1	0.03	592	507	0.89	0.16	39	472	±7	465	±19	-2	0.0563	0.8	0.59	1.8	0.076	1.6	0.9
28.1	0.00	158	149	0.97	0.26	25	1094	±15	1065	±17	-3	0.0749	0.8	1.91	1.7	0.185	1.5	0.9
29.1	0.58	78	27	0.36	0.50	3	259	±3	152	±148	-72	0.0491	6.3	0.28	6.4	0.041	1.3	0.2
30.1	0.08	98	84	0.88	0.34	13	931	±10	876	±29	-7	0.0682	1.4	1.46	1.8	0.155	1.2	0.6
31.1	--	195	72	0.38	0.33	26	942	±11	937	±17	-1	0.0703	0.9	1.52	1.5	0.157	1.3	0.8
32.1	0.07	284	130	0.47	0.47	14	369	±4	352	±33	-5	0.0535	1.4	0.43	1.9	0.059	1.2	0.7
33.1	--	804	746	0.96	0.14	55	490	±8	492	±14	+0	0.0570	0.6	0.62	1.8	0.079	1.7	0.9
34.1	0.03	109	60	0.57	0.36	18	1124	±19	1111	±20	-1	0.0766	1.0	2.01	2.1	0.191	1.9	0.9
35.1	0.12	80	37	0.48	6.77	5	415	±6	469	±62	+12	0.0564	2.8	0.52	3.2	0.066	1.5	0.5
36.1	--	258	147	0.59	0.25	13	359	±8	331	±31	-9	0.0531	1.4	0.42	2.8	0.057	2.4	0.9
37.1	0.12	168	41	0.25	1.37	8	366	±6	315	±68	-17	0.0527	3.0	0.42	3.5	0.058	1.8	0.5
38.1	0.01	861	84	0.10	2.70	131	1050	±18	1091	±18	+4	0.0758	0.9	1.85	2.0	0.177	1.8	0.9
39.1	0.02	750	324	0.45	0.17	28	278	±5	234	±22	-19	0.0508	1.0	0.31	1.9	0.044	1.7	0.9
40.1	0.07	548	79	0.15	0.77	37	485	±6	459	±19	-6	0.0562	0.9	0.61	1.5	0.078	1.3	0.8
41.1	0.08	215	128	0.62	0.62	22	730	±10	763	±23	+5	0.0646	1.1	1.07	1.8	0.120	1.4	0.8
42.1	0.04	633	356	0.58	0.40	25	288	±4	283	±24	-2	0.0519	1.1	0.33	1.8	0.046	1.4	0.8
43.1	0.25	225	203	0.93	1.20	63	1829	±18	2059	±13	+13	0.1271	0.7	5.75	1.4	0.328	1.1	0.8
44.1	0.01	189	257	1.40	0.42	45	1572	±34	1616	±39	+3	0.0996	2.1	3.79	3.2	0.276	2.4	0.8
45.1	0.00	93	44	0.49	0.43	4	340	±4	382	±87	+11	0.0543	3.9	0.41	4.0	0.054	1.2	0.3
46.1	0.12	304	104	0.35	0.29	16	387	±8	378	±37	-2	0.0542	1.7	0.46	2.7	0.062	2.2	0.8
47.1	0.34	1057	359	0.35	0.17	274	1697	±25	1976	±12	+16	0.1213	0.7	5.04	1.8	0.301	1.7	0.9
48.1	--	281	123	0.45	0.67	10	259	±4	263	±35	+1	0.0515	1.5	0.29	2.1	0.041	1.5	0.7
49.1	0.03	459	47	0.10	0.42	54	830	±9	787	±14	-6	0.0654	0.7	1.24	1.3	0.137	1.2	0.9
51.1	0.08	362	189	0.54	0.56	70	1304	±13	1377	±14	+6	0.0877	0.7	2.71	1.3	0.224	1.1	0.8
52.1	0.07	154	85	0.57	0.33	9	436	±5	441	±77	+1	0.0557	3.5	0.54	3.7	0.070	1.1	0.3
53.1	0.26	431	185	0.44	0.69	24	401	±4	381	±38	-5	0.0543	1.7	0.48	2.0	0.064	1.1	0.5
54.1	--	637	624	1.01	0.49	24	279	±4	290	±28	+4	0.0521	1.2	0.32	1.8	0.044	1.3	0.7
55.1	0.01	575	65	0.12	0.97	75	917	±54	829	±128	-11	0.0667	6.1	1.41	8.8	0.153	6.4	0.7
56.1	0.08	1317	679	0.53	0.32	72	397	±4	431	±16	+8	0.0555	0.7	0.49	1.3	0.064	1.0	0.8
57.1	0.00	435	60	0.14	0.33	128	1905	±21	1879	±6	-2	0.1150	0.3	5.45	1.3	0.344	1.3	1.0
58.1	0.38	61	36	0.60	0.48	3	365	±5	342	±113	-7	0.0533	5.0	0.43	5.2	0.058	1.3	0.3
59.1	0.18	86	51	0.61	0.41	19	1498	±16	1530	±22	+2	0.0951	1.1	3.43	1.7	0.262	1.2	0.7
60.1	0.50	570	261	0.47	0.19	20	256	±3	220	±54	-17	0.0505	2.3	0.28	2.7	0.041	1.4	0.5
61.1	0.12	111	66	0.62	1.46	5	324	±6	368	±66	+12	0.0539	2.9	0.38	3.4	0.052	1.8	0.5
62.1	0.01	562	80	0.15	0.28	94	1147	±14	1140	±8	-1	0.0777	0.4	2.09	1.4	0.195	1.3	1.0
63.1	--	213	121	0.59	0.26	28	927	±11	908	±16	-2	0.0693	0.8	1.48	1.5	0.155	1.2	0.8
64.1	--	13	1	0.04	3.73	2	1136	±21	1568	±104	+30	0.0970	5.5	2.58	5.9	0.193	2.0	0.3
65.1	0.03	438	286	0.67	0.19	61	964	±15	976	±12	+1	0.0717	0.6	1.59	1.8	0.161	1.7	0.9
66.1	0.03	660	217	0.34	0.33	88	930	±12	912	±10	-2	0.0694	0.5	1.49	1.4	0.155	1.4	0.9
67.1	0.04	123	50	0.42	0.38	14	823	±9	796	±26	-4	0.0657	1.2	1.23	1.7	0.136	1.1	0.7
68.1	0.03	1371	657	0.49	0.42	69	370	±4	386	±14	+4	0.0544	0.6	0.44	1.4	0.059	1.2	0.9
69.1	0.18	68	40	0.61	0.45	3	351	±7	376	±85	+7	0.0541	3.8	0.42	4.3	0.056	2.0	0.5
70.1	0.09	368	251	0.71	0.82	24	479	±5	488	±25	+2	0.0569	1.1	0.61	1.6	0.077	1.2	0.7

Error in Standard calibration was 0.27% (not included in above errors but required when comparing data from different mounts). (1) Common Pb corrected using measured 204Pb.

The zircon age distributions in all three samples show that they have a very similar provenance. These data also show that the jar 01020102 from Site 1 matches that from the sandstones and unfinished/abandoned jars from Site 21, suggesting this outcrop is the likely source of the material (Fig 13). Minor differences in the age profiles would be expected as the sandstones would show a natural variability. These data confirm that this technique provides excellent fingerprints of the megaliths and of their potential quarry sites, which are not always obvious nor proximal. Further analyses are being undertaken on other sites and will be used to match–or exclude–possible sources/quarries.

## Discussion

Archaeological research including excavation and survey conducted since 2016 has increased our understanding of the enigmatic megalithic culture of northern Laos. Through comparative typological analysis of the megaliths, similarities in morphology and stylistic treatment such as mouth shape, rim form [53] and general dimensions of the vessels at each of the sites investigated has been identified and would suggest contemporaneity of the sites [5,6]. The original purpose of the megalithic jars, however, remains a mystery. While Colani [2,3] reported finding human remains and glass beads in some megalithic jars, the mortuary purpose of the vessels remains to be confirmed through retrieval and dating of human remains found *in situ* preserved inside a jar.

With excavation we have established that the sites are associated with mortuary ritual, and have documented divergent mortuary practices at Site 1 comprising primary, secondary and ceramic burial jar interments [4,6]. Dating of the human skeletal material associated with these mortuary contexts, surrounding the jars, indicates that this activity occurred c. calAD 732–944, and latest at calAD 1043–1210. Whether the existence of primary burials and the two forms of secondary burials identified at Site 1 represents the ritual expression of a single culture or an additional temporal element requires further investigation. While similar funerary hallmarks such as buried limestone markers, chipped stone pavements and burial boulder markers were identified at Sites 2 and 52, no human skeletal material, aside from a single dental specimen, was uncovered to allow direct comparative dating of the burials.

The dating of the placement of the stone jars has, until recently, proven difficult not least because of the lack of associated organic material. A radiocarbon date obtained from charcoal from beneath one of the megalithic jars (W0018) at Site 2 and the OSL dates obtained from beneath two other jars (W0013 & W0021) at the same site, indicate that the jars were likely placed in their current location potentially as early as the late second millennium BC. A radiocarbon date obtained from charcoal from beneath one of the jars at Site 1 suggests a later event, in the 12th or 13th century AD [6]. This latter date must be viewed, as described above, with consideration of an adjacent burial which may post-date the emplacement of the megalithic jar.

The data presented here strongly suggests that the placement of the megaliths preceded the mortuary activity around the jars, indicating re-use of the sites and enduring ritual significance. While a human cranial sample obtained by Sayavongkhamdy (S1 Table) returned a date much earlier (2282–1265 calBC) than the three bone sample dates obtained by the authors at Site 1, which may indicate even earlier mortuary activity, this possibility requires further investigation. Very early dates were obtained from some charcoal samples taken from an observed ‘burning layer’ (c. 80cm below surface) noted in multiple areas across Site 1 (see S1 Table). This layer is not associated with any material culture.

The U-Pb dating undertaken on the zircons from a jar from Site 1 matches the dates obtained from rock and an unfinished jar at Site 21 (Phoukeng quarry), the presumed quarry. While this does not preclude that sandstone of similar age exists in other areas accessible to the site, it is the only known quarry site in close proximity to have been identified to date. Extensive geological mapping of the region is hindered by the lack of high-resolution maps and foot survey is not possible in many areas around the site due to UXO. How the jars, some estimated to weigh more than 30 tonnes, were transported from the quarry to their final position is unknown, though is likely to have required a substantial workforce. Phoukeng quarry is separated by 8 km of undulating terrain from Site 1. Whether the completed jars were dragged on some form of wooden rollers or sledge remains speculative.

## Conclusion

The megalithic sites of Laos have seen sporadic efforts in archaeological research since the 1930s, with more recent excavations at three of the main sites, namely Sites 1, 2 and 52. This research has created a deeper understanding of the prehistoric and historic cultures of Xieng Khouang Province. Dating for the placement of the jars and that of skeletal material found buried around the megaliths indicates multiple use of the sites and enduring ritual significance. While Colani [8] posits an Iron Age date for the creation of the sites, the dating of skull fragments obtained from investigations conducted by Sayavongkhamdy suggests burial activity at the sites from as early as 2282–1265 calBC. More recent analyses conducted by the authors (radiocarbon and OSL) suggests placement of the jars at one of the sites commenced potentially as early as the late second millennium BC, with ritual activity continuing into the historic period. Mortuary activity is noted at all three of the excavated sites based on similarities in the placement of limestone slabs over confirmed and suspected burials (soil acidity may have dissolved the bone at Sites 2 and 52). There are noted differences too as no ceramic jar burials, nor primary burials, were encountered at Sites 2 and 52 as they were at Site 1.

Although the original purpose of the megalithic jars remains to be determined, the present research indicates a long history of activity at the sites. The evidence provided by OSL dating has provided the first ever dates for the original placement of the jars at Site 2–1240 BC to 660 BC. While the broad similarity in megalith morphology across Laos might suggest contemporaneity and the expression of a unique, yet to be identified, cultural group, more research needs to be conducted. Future studies might usefully be directed at obtaining further samples from under, and at the sediment-artefact interface with the megalithic jars, at other sites and from across the geographic extent of the culture, using OSL to refine the earliest jar emplacement date. Efforts to ascertain the original purpose of the megaliths themselves should be directed at undisturbed sites or newly recorded sites and those with concealed contents or buried megalithic jars, instances of which are known.

## Supporting information

S1 Fig SEM cathodoluminescence (CL) images of representative zircons from the three samples analysed for this study. The locations of the SHRIMP spots are shown by the yellow ellipses and the labels refer to the data in the relevant tables.
(DOCX)

S1 Table Radiocarbon ages reported by Sayavongkhamdy* and Van Den Bergh^✝^ (2014) for Sites 1 and 2. (DOCX)

S2 Table Site 1 charcoal and bone dates from three excavation units. (DOCX)

S3 Table Numerical results of mixture modelling of U-Pb detrital zircon dates, showing the calculated components for each sample. *Relative misfit.
(DOCX)

S1 Text Zircon descriptions. (DOCX)
