# Supplementary material for: Dating the megalithic culture of laos: Radiocarbon, optically stimulated luminescence and U/Pb zircon results
Source: PLoS One. 2021 Mar 10;16(3):e0247167. doi: 10.1371/journal.pone.0247167 (PMC7946304; doi:10.1371/journal.pone.0247167)
Supplement: S1 Fig — The locations of the SHRIMP spots are shown by the yellow ellipses and the labels refer to the data in the relevant tables. (DOCX) [file pone.0247167.s001.docx]

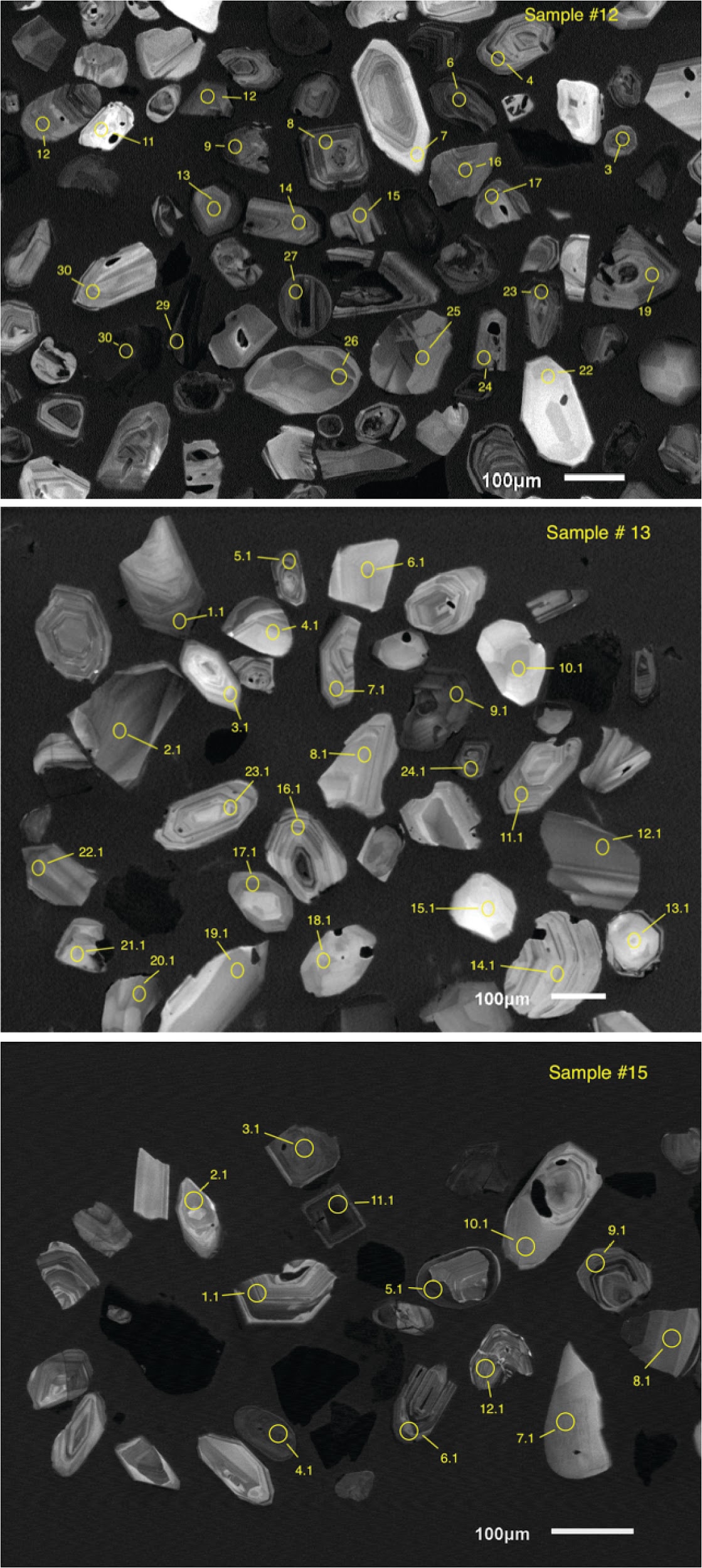


S1 Figure. SEM cathodoluminescence (CL) images of representative zircons from the three samples analysed for this study. The locations of the SHRIMP spots are shown by the yellow ellipses and the labels refer to the data in the relevant tables.
