# Supplementary material for: Dating the megalithic culture of laos: Radiocarbon, optically stimulated luminescence and U/Pb zircon results
Source: PLoS One. 2021 Mar 10;16(3):e0247167. doi: 10.1371/journal.pone.0247167 (PMC7946304; doi:10.1371/journal.pone.0247167)
Supplement: S1 Table — (DOCX) [file pone.0247167.s002.docx]

| Provenance | lab number | Material | Depth below surface (m) | ^14^C yrs BP | Calibrated Date (IntCal20, 95% CI) BC/AD |
| --- | --- | --- | --- | --- | --- |
| Site 1 Group 2 | OZD-770* | Bone | 0.79 | 3410 ±190 | 2282–1265 calBC |
| Site 1 Group 2 | ANU10767* | Charcoal | 0.29 | 920 ± 50 | 1027–1220 calAD |
| Site 1 Group 2 | ANU10764* | Charcoal | 0.8 | 8320 ± 100 | 7577–7079 calBC |
| Site 1 Group 2 | ANU10765* | Charcoal | 0.79 | 8270 ± 120 | 7577–7049 calBC |
| Site 1 Group 2 | ANU10766* | Charcoal | 0.72 | 8150 ± 90 | 7468–6827 calBC |
| Site 2 burial pit | AA81046^✝^ | Charcoal | 0.37 | 1034 ± 37 | 895-1151 calAD |
| Site 1 burial | AA6146^✝^ | Charcoal | 0.35 | 935 ± 50 | 1025–1215 calAD |
| Site 1 burial pit | AA6147^✝^ | Charcoal | 0.58 | 4430 ± 50 | 3335–2919 calBC |

**S1 Table**. Radiocarbon ages reported by Sayavongkhamdy* and Van Den Bergh^✝^ (2014) for Sites 1 and 2.

For ease of comparison with current dating programme results, these previously reported radiocarbon results have been calibrated using the IntCal 20 atmospheric calibration curve [Brock et al. 2010; Ramsey 2017; Reimer et al. 2020]. The analyses were undertaken at ANU and the University of Arizona.
