# Supplementary material for: Dating the megalithic culture of laos: Radiocarbon, optically stimulated luminescence and U/Pb zircon results
Source: PLoS One. 2021 Mar 10;16(3):e0247167. doi: 10.1371/journal.pone.0247167 (PMC7946304; doi:10.1371/journal.pone.0247167)
Supplement: S2 Table — (DOCX) [file pone.0247167.s003.docx]

| Provenance | Lab number | Material | Depth below surface (m) | ^14^C yrs BP | Calibrated Date  (Confidence 95.4%) |
| --- | --- | --- | --- | --- | --- |
| U1 Burial 2 | ANU49220 | Charcoal | 0.11 | 984 ± 26 | 994–1157 calAD |
| U1 Burial 2 | ANU49221 | Charcoal | 0.11 | 861 ± 31 | 1050-1265 calAD |
| U1 Burial 2 | ANU49235 | Charcoal | 0.11 | 965 ± 24 | 1025-1158 calAD |
| U1 Burial 2 | ANU62918 | Bone | 0.11 | 896 ± 22 | 1046-1219 calAD |
| U1B (extension of unit called 1B) Burial 3 | ANU49231 | Charcoal | 0.16 | 1036 ± 24 | 977-1035 calAD |
| U1 (extension of unit called 1B) Burial 3 | ANU49232 | Charcoal | 0.16 | 1074 ± 25 | 894-1024 calAD |
| U1 1:2  layer 1 spit 2 | ANU49233 | Charcoal | 0.16 | 985 ± 24 | 945-1155 calAD |
| U1 1:3  layer 1 spit 3 | ANU49218 | Charcoal | 0.23 | 932 ± 27 | 1032-1175 calAD |
| U1 1:3  layer 1 spit 3 | ANU49219 | Charcoal | 0.23 | 960 ± 25 | 1027-1158 calAD |
| U1 1:3  layer 1 spit 3 | ANU49228 | Charcoal | 0.26 | 866 ± 30 | 1050-1262 calAD |
| U1 1:3  layer 1 spit 3 | ANU49223 | Charcoal | 0.26 | 972 ± 26 | 1022-1158 calAD |
| U1 1:3  under jar 01020061 | ANU49227 | Charcoal | 0.26 | 838 ± 24 | 1168-1264 calAD |
| U1 1:4  layer 1 spit 4 | ANU49229 | Charcoal | 0.32 | 1000 ± 24 | 992-1151 calAD |
| U1 2:2  layer 2 spit 2 | ANU49224 | Charcoal | 0.51 | 4389 ± 29 | 3093-2916 calBC |
| U1 2:2  layer 2 spit 2 | ANU49225 | Charcoal | 0.51 | 4400 ± 31 | 3308-2912 calBC |
| U1 Sondage (deep pit within Unit 1) | ANU49226 | Charcoal | 0.78 | 1165±26 | 772-975 calAD |

| U2  Layer 2 spit 2 | ANU49237 1008 ± 25  Combined with duplicate  ANU49320 997 ± 24    R_Combine 95.4% probability 1002 ± 18  X2-Test: df=1 T=0.1 (5% 3.8) | Charcoal | 0.46 | 1002 ± 18 | 991-1147 calAD |
| --- | --- | --- | --- | --- | --- |
| U2  Layer3, spit 1 | ANU49238 | Charcoal | 0.49 | 888 ± 26 | 1046-1223 calAD |
| U2  Layer 3, spit 1 | ANU49230 | Charcoal | 0.49 | 4037 ± 28 | 2629-2470 calBC |
| U2  Layer 3, spit 1 | ANU49236 | Charcoal | 0.49 | 4077 ± 31 | 2856-2491 calBC |
| U2  Layer 3, spit 2 | ANU49316 | Charcoal | 0.73 | 1007 ± 24 | 991-1149 calAD |
| U2 Burial 5 | ANU49317 | Charcoal | 0.81 | 1091 ± 25 | 892-101612 calAD |
| U2 Burial 7 | ANU49318 | Charcoal | 0.90 | 1069 ± 25 | 895-1025 calAD |
| U2 Burial 5/7 | ANU49306 | Charcoal | 0.97 | 1097 ± 29 | 889-1016 calAD |
| U2 Burial 5 | ANU49319 | Charcoal | 0.97 | 1075 ± 24 | 895-1023 calAD |
| U2 Burial 5 | ANU53420 | Bone | 0.97 | 1036 ± 23 | 978-1035 calAD |

| U3 Burial 4 | ANU49307 956 ± 24  combined with duplicate ANU49315 980 ± 25    R_Combine 95.4% probability 968 ± 18  X2-Test: df=1 T=0.5 (5% 3.8) | Charcoal | 0.28 | 968 ± 18 | 1026-1155 calAD |
| --- | --- | --- | --- | --- | --- |
| U3 Burial 4 | 28 | Charcoal | 0.28 | 954 ± 28 | 1029-1159 calAD |
| U3  Layer 3, spit 1 | ANU49310 | Charcoal | 0.51 | 8717 ± 35 | 7939-7598 calBC |
| U3  Layer 3, spit 2 | ANU49312 | Charcoal | 0.65 | 8850 ± 36 | 8210-7794 calBC |
| U3 Burial 6 | ANU49313 | Charcoal | 0.76 | 46791 ± 3188 | 59623–39939 calBC* |
| U3  Layer 3, spit 3 | ANU49314 | Charcoal | 0.77 | 8606 ± 35 | 7731-7579 calBC |
| U3 Burial 6 | ANU62919 | Bone | 0.92 | 1185 ± 27 | 772-950 calAD |

* This result (ANU49313) is discordant with dates from a similar depth. It is not included in Figure 9.

**S2 Table.** Site 1 charcoal and bone dates from three excavation units.

The dates presented have previously been published in O’Reilly et al. (2019) but are here recalibrated using OxCal 4.4 and the IntCal 20 atmospheric calibration curve [Brock et al. 2010; Ramsey 2017; Reimer et al. 2020] and presented according to depth below surface.
