# Supplementary material for: Dating the megalithic culture of laos: Radiocarbon, optically stimulated luminescence and U/Pb zircon results
Source: PLoS One. 2021 Mar 10;16(3):e0247167. doi: 10.1371/journal.pone.0247167 (PMC7946304; doi:10.1371/journal.pone.0247167)
Supplement: S3 Table — *Relative misfit. (DOCX) [file pone.0247167.s004.docx]

| **Sample #12** | | **Sample #13** | | **Sample #15** | |
| --- | --- | --- | --- | --- | --- |
| Ages (Ma) | ±2s | Ages (Ma) | ±2s | Ages (Ma) | ±2s |
| 268 | 2 | 272 | 2 | 273 | 2 |
| 335 | 4 | 336 | 3 | 335 | 7 |
| 370 | 6 | 390 | 7 | 364 | 4 |
| 419 | 5 | 439 | 7 | 411 | 4 |
| 473 | 7 | 485 | 5 | 471 | 5 |
| *0.077** | | *0.074** | | *0.070** | |

| S3 Table: Numerical results of mixture modelling of U-Pb detrital zircon dates, showing the calculated components for each sample. **Relative misfit* |
| --- |

**References**

Brock F, Higham T, Ditchfield P, Ramsey CB. Current pretreatment methods for AMS radiocarbon dating at the Oxford Radiocarbon Accelerator Unit (ORAU). Radiocarbon. 2010;52(1):103-12.

O'Reilly D, Shewan L, Domett K, Halcrow SE, Luangkhoth T. Excavating among the megaliths: recent research at the ‘Plain of Jars’ site 1 in Laos. Antiquity. 2019 Aug;93(370):970-89.

Ramsey CB. Methods for summarizing radiocarbon datasets. Radiocarbon. 2017 Dec;59(6):1809-33.

Reimer P, Austin W, Bard E, Bayliss A, Blackwell P, Bronk Ramsey C, Butzin M, Cheng H, Edwards R, Friedrich M, Grootes P, Guilderson T, Hajdas I, Heaton T, Hogg A, Hughen K, Kromer B, Manning S, Muscheler R, Palmer J, Pearson C, van der Plicht J, Reimer R, Richards D, Scott E, Southon J, Turney C, Wacker L, Adolphi F, Büntgen U, Capano M, Fahrni S, Fogtmann-Schulz A, Friedrich R, Köhler P, Kudsk S, Miyake F, Olsen J, Reinig F, Sakamoto M, Sookdeo A, & Talamo, S. The IntCal20 Northern Hemisphere radiocarbon age calibration curve (0–55 cal kBP). Radiocarbon. (2020) 62 (4):725-757. https://doi.org/10.15184/aqy.2019.102.

Van Den Bergh J and Luangaphay S. Heritage Management at Plain of Jars Archaeological Landscape (unpublished). Presented at the Sogang Institute for East Asian Studies International Cluster Conference *Problematising Megaliths of Southeast Asia and the Pacific*, 2014 Sogang University, Korea.
