# Supplementary material for: Dating the megalithic culture of laos: Radiocarbon, optically stimulated luminescence and U/Pb zircon results
Source: PLoS One. 2021 Mar 10;16(3):e0247167. doi: 10.1371/journal.pone.0247167 (PMC7946304; doi:10.1371/journal.pone.0247167)
Supplement: S1 Text — (DOCX) [file pone.0247167.s005.docx]

S1 Text: Zircon descriptions

The zircons separated from each of the three samples are heterogeneous in terms of size, shape and form. This was not unexpected considering they were all sourced from sandstones with very similar mineralogy, grain size and appearance. Most of the grains are fragments and show rounding and abrasion from the weathering, erosion, transportation and deposition processes they were subjected to. Some are highly rounded and may represent survivors of secondary or more deposition cycles. A number are euhedral to subhedral, clear and have not been significantly abraded and could be from proximal sources. Cathodoluminescence (CL) imaging of the grains show a full spectrum from highly zoned (magmatic) to featureless grains with no zoning. Core-rim structures are present, but not common. A compilation of representative CL images is shown in S1 Figure.
